# Resting-State Functional Connectivity in the Infant Brain: Methods, Pitfalls, and Potentiality

**DOI:** 10.3389/fped.2017.00159

**Published:** 2017-08-14

**Authors:** Chandler R. L. Mongerson, Russell W. Jennings, David Borsook, Lino Becerra, Dusica Bajic

**Affiliations:** ^1^Center for Pain and the Brain, Boston Children’s Hospital, Boston, MA, United States; ^2^Department of Anesthesiology, Perioperative and Pain Medicine, Boston Children’s Hospital, Boston, MA, United States; ^3^Department of Surgery, Boston Children’s Hospital, Boston, MA, United States; ^4^Department of Surgery, Harvard Medical School, Boston, MA, United States; ^5^Department of Anaesthesia, Harvard Medical School, Boston, MA, United States

**Keywords:** blood oxygen level dependent, functional magnetic resonance imaging, independent component analysis, magnetic resonance imaging, resting-state functional magnetic resonance imaging, neurodevelopment, pediatric, resting-state networks

## Abstract

Early brain development is characterized by rapid growth and perpetual reconfiguration, driven by a dynamic milieu of heterogeneous processes. Postnatal brain plasticity is associated with increased vulnerability to environmental stimuli. However, little is known regarding the ontogeny and temporal manifestations of inter- and intra-regional functional connectivity that comprise functional brain networks. Resting-state functional magnetic resonance imaging (rs-fMRI) has emerged as a promising non-invasive neuroinvestigative tool, measuring spontaneous fluctuations in blood oxygen level dependent (BOLD) signal at rest that reflect baseline neuronal activity. Over the past decade, its application has expanded to infant populations providing unprecedented insight into functional organization of the developing brain, as well as early biomarkers of abnormal states. However, many methodological issues of rs-fMRI analysis need to be resolved prior to standardization of the technique to infant populations. As a primary goal, this methodological manuscript will (1) present a robust methodological protocol to extract and assess resting-state networks in early infancy using independent component analysis (ICA), such that investigators without previous knowledge in the field can implement the analysis and reliably obtain viable results consistent with previous literature; (2) review the current methodological challenges and ethical considerations associated with emerging field of infant rs-fMRI analysis; and (3) discuss the significance of rs-fMRI application in infants for future investigations of neurodevelopment in the context of early life stressors and pathological processes. The overarching goal is to catalyze efforts toward development of robust, infant-specific acquisition, and preprocessing pipelines, as well as promote greater transparency by researchers regarding methods used.

## Introduction

### Definition of Method

Functional magnetic resonance imaging (fMRI) has evolved into an important non-invasive neuroinvestigative tool, used to probe underlying systems-level mechanisms in the brain. Specifically, fMRI provides an indirect measure of brain activity by detecting fluctuations in blood flow and oxygenation, referred to as blood oxygen level dependent (BOLD) effect (1). Interpretation of fluctuations in BOLD signal derives from the physiological concept of neurovascular coupling, whereby neuronal activity and regional cerebral blood flow are tightly linked (2–4). Resting-state functional magnetic resonance imaging (rs-fMRI) examines spontaneous low frequency fluctuations in brain activity (5) present during physiological (sleep), pharmacological (anesthesia, sedation), and clinically induced (e.g., coma) unconscious states (6). Specifically, it describes intrinsic brain activity in its “resting state”—a key departure from classical task-based fMRI. Mapping temporal covariance in brain activity between distinct brain regions (i.e., functional connectivity) reveals correlated patterns of large-scale neural networks, termed resting-state networks (RSNs) (7, 8). These RSNs encompass brain regions that are anatomically linked and known to mirror functional networks activated during task-oriented behaviors (9–12). Furthermore, this intrinsic brain activity predicts task performance and likely contributes to behavioral variability (13). With more than a decade’s head start, rs-fMRI in adults has begun to mobilize efforts toward standardized methods for optimal data acquisition and processing. However, its application in neonates and infants is only just emerging, with little or no reported infant-specific preprocessing measures. Therefore, we describe a robust method to perform RSN analysis in neonates and infants, and discuss challenges associated with rs-fMRI application in this population.

### RSNs in Developing Brain

Resting-state functional magnetic resonance imaging derives its growing popularity from its unique method of acquisition (i.e., limited need for subject participation), enabling application in expanded patient populations previously unsuitable for task-based fMRI. Until recently, patient motion during scanning precluded robust functional interrogations of the infant brain using fMRI, with few exceptions (14–20). Rs-fMRI holds considerable appeal for studies of infants and early brain development, compared to task-based fMRI. Scans acquired during natural sleep in the absence of experimental stimuli eliminate many of the confounding variables associated with appropriate paradigm selection, including network-specific reactivity to distinct stimuli, as well as age- and/or cognitive-level-dependent responses (21). Such variables often impede extrapolation of findings to other age groups, critical for investigations of longitudinal neurodevelopment. Over the past decade, the emergence of infant rs-fMRI offered insight into patterns of functional connectivity, yielding more complete representations of neural networks and their development. First described in Fransson’s seminal paper (22), the presence of RSNs in infants have been established as early as the fetal (23, 24), preterm (22, 25, 26), and infant periods (27–29), undergoing substantial maturation and refinement over the first two decades of life (30–34). Despite limited published literature since its inception in 2007, significant groundwork has been laid in the field of infant rs-fMRI, offering transient glimpses into the complex interplay of structural and functional brain development.

### Review of Early Brain Development

Brain development is a continuous process initiated during early gestation that extends into postnatal life and beyond into adolescence (35, 36). Evolving cerebral architecture matures at different rates, establishing critical periods for development of specific functions. The brain’s capacity for dynamic adaptation and reorganization through neural plasticity is a function of heterogeneous mechanisms, which work to optimize integration of various functions (e.g., sensory, motor, cognitive). For example, ongoing synaptic plasticity involves a developmental balance between synaptogenesis (formation) and pruning (elimination) of synapses in the brain (37). These processes influence the survival of specific neural circuits contributing to the formation, reconfiguration, and maturation of diverse, complex functional brain networks. In contrast, longitudinal studies probing the exact timing and evolution of RSNs maturation to adult patterns are scarce, contributing to an incomplete understanding of postnatal neurodevelopment. Moreover, investigations to date have primarily centered around relatively *healthy* preterm and term infants to establish normative patterns of resting-state activity during early development. Targeted studies to understand the potential effects of certain clinical treatments (e.g., surgery, drug exposure, pain management), as well as to model neurodevelopmental progression of diseases/disorders in infant and pediatric populations remain largely unexplored due to current methodological limitations of rs-fMRI analysis at this early age. These challenges are discussed at length throughout the method, as well as later in the *Discussion* section.

### Resting-State Network Analysis Techniques

Standard methods used to analyze rs-fMRI data include independent component analysis (ICA) (38, 39) and seed-based correlation analysis (SCA) (7, 40). Despite distinct analytical approaches, both techniques result in identification of comparable RSNs (41). SCA is a model-based, *hypothesis-driven* approach used to measure BOLD response in an anatomically defined seed region-of-interest (ROI), ultimately generating whole-brain correlation maps. This technique is optimal when testing a very specific hypothesis, using an explicit temporal model derived from predetermined seed region (39). Though simple for probing brain activity between various conditions (e.g., patient versus control), placing ROI with anatomical specificity and accuracy is more challenging and, at this time, still requires manual outlining of the anatomical structure in the infant brain. An additional limitation of ROI-based analyses stems from the inherent bias introduced by selection of seed regions, which precludes characterization of whole-brain functional connectivity. Alternatively, ICA is a model-free, *data-driven* approach, ideal for exploratory analysis and/or in cases where no suitable hypothesis is available (42). Based on the widely used multivariate statistical technique, principal component analysis (PCA), ICA decomposes data into components with maximal statistical independence in the spatial domain (39). Probabilistic ICA (PICA) has since evolved, specifically tailored for use in fMRI inquiries (43). ICA model is also uniquely advantageous in that inherent data transformations therein necessarily produce several artifactual components in every ICA output (11). Accordingly, ICA, in addition to its role during final analyses, can also be employed as part of data preprocessing to isolate sources of non-neuronal noise (44).

In summary, rs-fMRI and ICA combined provide a non-invasive, neuro-investigatory technique for reliable extraction of brain networks, ideal for use in infants due to the inherent method of acquisition (i.e., non-task-based, performed during sleep). Although this methodology is not novel, there are currently no available protocols for performing RSN analysis in the infant brain. Moreover, this burgeoning field has yet to establish standardized methodological pipelines for infant populations. We aim to provide a detailed, albeit basic method for RSN analysis in infants, with interwoven discussions of theoretical background and parameter selections for each technique. If nothing more, this method may serve as a proof-of-concept, underscoring the need for systematic, infant-specific methodologies necessary to drive rs-fMRI possibly into routine clinical practice.

## Methodological Outline

Infant data used to illustrate steps in this protocol was acquired as part of a larger investigational study that was reviewed and approved by the Boston Children’s Hospital Institutional Review Board and classified as a no more than minimal risk study. The study also conformed to the standards set by the Declaration of Helsinki and Good Clinical Practice guidelines. Eligibility criteria included absence of magnetic resonance imaging (MRI) incompatible implants. Informed written consents were obtained from parents before MRI scans were collected. Detailed approaches as to how to safely handle non-sedated infants for MRI acquisitions are available (45–47), however, are beyond the scope of current manuscript.

### Equipment

#### MRI Scanner

We used 3T MRI scanner (Trio Tim, Siemens Medical Solutions USA, Inc., Malven, PA, USA) equipped with a 32-channel receive-only head coil.

#### Computing Hardware for Analysis

Unix-based computer is recommended for computation, as FMRIB Software Library (FSL) programming is precompiled for Mac users (Mac OS X 10.4 or higher), as well as PC users running Linux virtual machines (i.e., RedHat 9, Centos, Debian/Ubuntu) (48). The computer itself should have at minimum a “1 GHz CPU clock, 1 GB RAM, 5 GB sway, and 20 GB of free hard drive space” if it is to be used for the analysis. Computer clusters (multiple computers networked together that act as a single, more powerful unit) are useful in reducing time penalties associated with analysis.

#### Computing Software Needed for the Analysis

- Terminal window, also referred to as a terminal emulator, is a text-only window in a graphical user interface (GUI) that emulates a *console*.
- MATLAB (The MathWorks Inc.^1^).
- Software packages like dcm2nii and MRIcron for preprocessing and network visualization, respectively.^2^
- FMRIB Software Library is freely available software from the Analysis Group at the University of Oxford.^3^ Within FSL, ICA can be performed using the Multivariate Exploratory Linear Decomposition into Independent Components (MELODIC) interface.^4^ Melodic uses ICA to break down 4D (3D-space and time) datasets into distinct spatial and temporal components. Specifically, ICA is a computational method that separates a multivariate signal into components, which are assumed to be statistically independent of one another. Brain extraction tool (BET) (49) within FSL is also required.
  - Alternative software exists that will allow for functional connectivity analysis comparable to that described in method [e.g., the Group ICA of fMRI Toolbox (GIFT) ICA package in MATLAB^5^].

### MRI Acquisition

Optimized rs-fMRI data acquisition parameters are vital for obtaining quality images that allow for more reliable and robust analyses. Reports on optimal scan parameters in infants are beginning to emerge (50). For current recommended guidelines to obtain high-quality MRI images in adults, refer to report by Smith et al. (44). Two types of scans are necessary to perform rs-fMRI analysis in the present protocol:

- High-resolution structural MR images. Parameters used to acquire T1-weighted sagittal sequence [also referred to as magnetization prepared rapid acquisition gradient echo (MPRAGE) image] of representative infants in our study were as follows: repetition time (TR) 2,520 ms; echo time (TE) 1.75 ms; field of view 180 mm × 180 mm; 144 slices; voxel size 1.0 mm × 1.0 mm × 1.0 mm.
- Resting-state functional MR images. Parameters used to acquire multiband gradient echo echo-planar imaging (EPI) rs-fMRI sequence of representative infants in our study were as follows: TR 1,830 ms; TE 36 ms; field of view 160 mm × 160 mm; 63 slices; voxel size 2.0 mm × 2.0 mm × 2.0 mm; flip angle 65^o^.

### Procedural Outline

Resting-state functional magnetic resonance imaging is a very complicated imaging technique with respect to physics, physiological mechanisms, data analysis, and interpretation. We provide a comprehensive step-by-step methodological outline such that investigators without previous knowledge in the field can implement the analysis and reliably obtain viable results consistent with previous literature. Specifically, we provide detailed, albeit basic methodological framework for RSN analysis, with interwoven discussion of basic theory behind each step, as well as the rational behind selecting parameters. Resting-state network analysis can be divided into three major parts: (I) data quality evaluation (see Method Part I. Data Quality Assessment), (II) preprocessing (see Method Part II. Preprocessing), and (III) analysis (see Method Part III. Final Analysis). Following initial fMRI data quality assessment, subsequent preprocessing stage entails a series of steps aimed at preparing structural and functional images for final analysis using single-subject single-session PICA. The latter is also employed during preprocessing to perform the relatively new automated method of ICA-based artifact removal using FMRIB’s ICA-based X-noiseifier (FIX) (44). Finally, we outline steps and differences between single-session versus group analysis, as well as interpretation and presentation of the rs-fMRI data.

## Method Part I. Data Quality Assessment

### Initial Step: Convert All Raw Files to Usable Format

Raw imaging data collected straight from the scanner are in dicom format (.dcm). This format is incompatible with many of the analytical tools used during preprocessing and final analyses described below, necessitating conversion of raw dicom files to nifti format (.nii.gz; high dynamic range image file <hdrfile>). This can be accomplished using the software dcm2nii. To convert files intended for analysis (both structural and functional), one should click and drag the folder containing the dicom images into the dcm2nii GUI.

### Time-Series Length Criterion

As a first step, one should ascertain the length of the fMRI BOLD time-series (e.g., 280 volumes). Total number of volumes can be obtained by opening the functional image in FSLView and scrolling down to “Cursor Tools” panel (see also https://fsl.fmrib.ox.ac.uk/fsl/fslwiki/FslView/UserGuide). Determining total number of volumes provides information on whether the infant’s scan ran to completion and ensures each file included in subsequent analysis contains sufficient data to accurately reflect subject’s BOLD signal activity (e.g., >200 volumes was arbitrarily selected in current protocol). Ultimately, optimal volume criterion will vary depending on the quantity (number of volumes acquired) and quality (e.g., image resolution or presence of artifact) of fMRI data collected, as well as the type of analysis you wish to perform [e.g., single-session or group ICA (GICA); see Final Statistical Analysis Using ICA in Section “Final Analysis”]. For adult subjects, general consensus in the resting-state field typically requires at least 5 min of useable data post preprocessing in adult populations to achieve brain network stability (41, 51). However, minimum scan length necessary to properly characterize RSNs in the infant brain has not been established.

Additional measures for quality assessment of infant fMRI data should be carried out prior to subsequent processing steps. This study used the Biomedical Informatics Research Network (BIRN) human quality assessment tool (52), implemented in MATLAB. Numerous measures of signal quality are analyzed, including: average signal intensity, radius of decorrelation (53, 54), and signal-to-fluctuation-noise ratio, as well as percent fluctuation, drift, and peak-to-peak amplitude size of the BOLD signal in fMRI data. Though an understanding of measures used to assess data quality is important, this lies beyond the scope of current protocol.

## Method Part II. Preprocessing

Preprocessing stage of analysis involves processing of both structural and functional images. Rather than performing individual preprocessing steps separately in terminal, GUIs are implemented wherever possible to compartmentalize preprocessing pipeline. Structural MR images are prepared first to create a reference image necessary for subsequent functional data preprocessing (prerequisite for ICA). Preprocessed versions of structural and functional data are used for final analysis.

### Preprocessing Structural MRI Data

**Figure 1:**
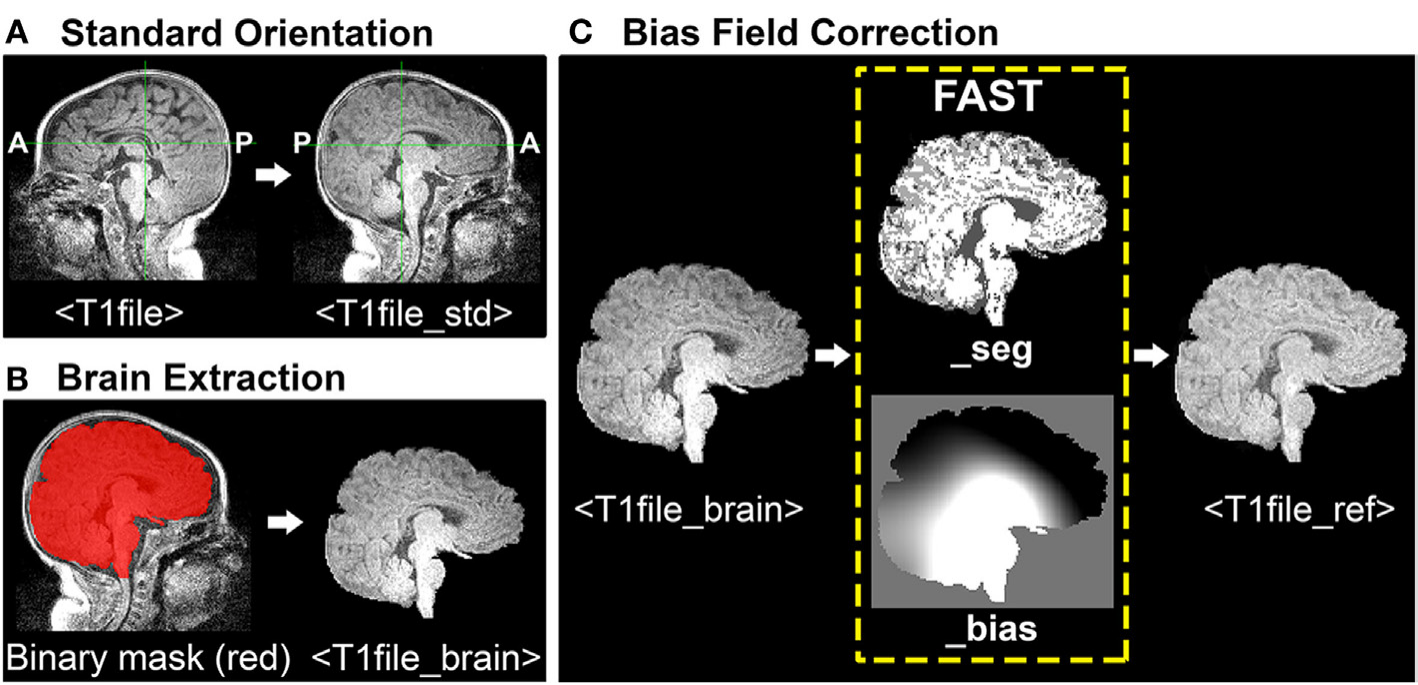
Structural magnetic resonance imaging (MRI) data preprocessing pipeline. Figure illustrates structural MRI data preprocessing pipeline, using a representative ex-33 week premature infant scanned at 4-weeks corrected age (2.75 months postnatal age). Panel **(A)** shows conversion of original T1-weighted image (*T1file*, in sagittal view) to a standardized brain orientation (*T1file_std*). Note, anterior (A) and posterior (P) are flipped in reoriented file. Panel **(B)** illustrates process of brain extraction. Manually edited binary mask (*T1file_bet_mask*; red) is shown superimposed on T1 image (grayscale), as well as finalized extracted brain (*T1file_brain*). Panel **(C)** illustrates the process of bias field correction. FMRIB’s Automated Segmentation Tool tissue-type segmentation (*_seg*) and estimated bias field (*_bias*) output files are shown. The latter image reflects gradient of tissue-specific signal inhomogeneity, showing hypo- (black) and hyper-intense (white) regions across entire T1 image. Hyperintense subcortical regions present in original T1 image are attenuated following intensity normalization, creating finalized structural Reference image (*T1file_ref*).

During ICA, each functional image included in analysis is registered (spatially aligned) to its corresponding structural image (i.e., its native space), often referred to as the Reference image. When selecting a Reference image, one should choose the anatomical image exhibiting the best resolution and tissue contrast for optimal registration results. Poor tissue contrasts is very common during the first year of life, catalyzing recent efforts to develop robust neonatal tissue segmentation techniques (55). This protocol uses structural T1-weighted images (although T2-weighted scans may also be used, and oftentimes exhibit better tissue contrasts compared to T1 images). To generate a structural Reference image for registration, the following three steps are required: (1) re-orientation and verification of header information, (2) brain extraction, and (3) bias field correction (i.e., intensity normalization). See Figure 1 for illustrative pipeline.

#### Orientation of Images

Structural T1-weighted images (*viz*. MPRAGE) must have correct header information (anterior-posterior, left-right, superior-inferior) to be correctly processed in subsequent analysis using FSL. To verify accurate header information, one should visualize each structural file in FSLView with the *fslview* command: *fslview* <T1file>.

Images should be uniformly oriented according to *radiological* convention (left side of image corresponds to right side of brain) in accordance with FSL’s common coordinate system [e.g., adult atlas Montreal Neurological Institute (MNI) 152 standard-space (57)]. This is in contrast to brain images oriented in *neurological* convention (left side of image corresponds to left side of brain). To re-orient image, one should use the *fslreorient2std* command as follows: *fslreorient2std* <T1file><T1file_std>.

This command does not “register” the T1 image to MNI152 standard-space. It simply rotates or flips images on the three axes so that orientation labels correspond to the standard template (Figure 1A). Finally, one should verify that T1 image was properly oriented by visualizing file in FSLView using the *fslview* command.

#### Brain Extraction

Brain Extraction Tool (BET) is an algorithm originally developed for the adult brain that allows for removal of non-brain voxels from MR images (e.g., T1 image) (49). However, application in infants is less reliable and often results in a rough outline of the infant brain, necessitating further manual editing to establish an accurate brain outline. For a detailed review of challenges associated with neonatal brain segmentation, and alternative methods currently available, refer to recent review (58). The overarching goal of manually editing automated BET binary mask file is to ensure inclusion of all brain tissue, while minimizing inclusion of all non-brain tissue (Figure 1B). This can be challenging when addressing infant MRI scans, which often display tissue inhomogeneity, as well as gray-matter–white-matter reversal. Moreover, infant brains are known to exhibit a high degree of morphological variation, particularly in the setting of rapid postnatal development. As such, individuals responsible for brain extraction should understand MR signal characteristics of different tissue densities, and be familiar with neuroanatomy and surrounding structures (e.g., subarachnoid cisterns, dural venous sinuses, cranial architecture). Deficits in either capacity will likely result in non-brain tissue inclusion, leading to suboptimal structural-to-functional image alignment during registration.

To perform brain extraction on T1 image and generate a binary mask file (for the purposes of manual editing), use the *bet* command as follows: *bet* <T1file_std> <T1file_bet> -c <x y z> -m.

Option –c designates the center of the brain coordinates (i.e., *x, y, z*; in voxels), which helps guide brain extraction. Option –m instructs program to generate a binary mask file containing extracted brain (*T1file_bet_mask*). Automated binary mask file can be edited within FSLView, using pencil and eraser tools to draw in missing brain tissue and remove non-brain tissue, respectively. The original T1 image (*T1file_std*) should also be opened to provide anatomical context and guide manual tracing of brain outline. To simultaneously view the binary mask file overlaid on original T1 image, one should use the following command: *fslview* <T1file_std> <T1file_bet_mask> -l Red.

Option –l Red will change color of binary mask file to red, allowing the viewer to distinguish the mask file from underlying original T1 image in grayscale (Figure 1B). Once editing is complete, apply manually edited binary mask file (includes brain only) to the original T1 image (includes brain and surrounding tissue) by using the following command: *fslmaths* <T1file_std> –mas <T1file_bet_mask> <T1file_brain>.

This creates the finalized brain extracted image (*T1file_brain*), required for bias field correction by FAST (next step).

#### Bias Field Correction

Bias fields refer to intensity inhomogeneity across magnetic resonance images (Figure 1C). Strong bias fields can cause serious mislabeling of voxel tissue-types, compromising accuracy of techniques that rely heavily on tissue densities (e.g., registration), and in particular gray and white matter contrast (21). FMRIB’s Automated Segmentation Tool (FAST) is a fully automated, robust, and reliable method for simultaneous tissue-type segmentation and bias field estimation, available within FSL (59). Specifically, FAST assigns each voxel a specific tissue-type (based on estimated class mean intensities and labeling of neighboring voxels), after which tissue-specific intensity inhomogeneity is evaluated (59). Bias field correction requires initial generation of estimated bias field using FAST, followed by normalization of signal intensity (Figure 1C). To open FAST GUI, type “*Fast*” into Linux terminal. Once interface opens, instructions to generate the estimated bias field are as follows:

- Input options. Select the number of structural T1 images to be assessed under “number of input channels”, and then input desired images (e.g., *T1file_brain*). Input files must already be brain extracted. Select T1-weighted for “Image type.” Select T2-weighted for “Image type”, or select T2-weighted if desired input file is either a T2-weighted or fMRI image.
- Output options. Leave “number of classes” (i.e., tissue-types) to be segmented at the default setting. This instructs FAST to segment T1 image into three tissue classes [gray matter, white matter, and cerebrospinal fluid (CSF)]. However, cases will arise when it may be appropriate to alter class number. For instance, neonatal MR images will occasionally exhibit extremely poor gray-matter–white-matter contrast due to incomplete myelination during postnatal period. Depending on severity, such an image may require selection of only two tissue classes, such that FAST segments image into brain tissue (i.e., combined gray/white matter) and CSF. Blending of tissues should be avoided, however, as this will have implications for subsequent processing steps that rely on such tissue contrasts [e.g., registration using boundary-based registration (BBR) algorithm; see Use Melodic for fMRI Data Preprocessing in Section “Preprocessing”].

Once FAST interface setup is finished, press “Go” to run the analysis. FAST output includes two files, ending with “_seg” (*T1file_brain_seg*) and “_bias” (*T1file_brain_bias*). The latter shows the estimated bias field, evaluating regions of intensity inhomogeneity across the entire T1 image. To normalize intensity values (i.e., perform bias field correction), use the following command in FSL terminal: *fslmaths* <T1file_brain> –div <T1file_brain_bias> <T1file_ref>.

Option –div instructs program to divide brain extracted T1 image by its bias field, thereby normalizing intensity distribution for each tissue class. Output file (*T1file_ref*) will serve as the Reference image during registration.

### Preprocessing Functional MRI Data

**Figure 2:**
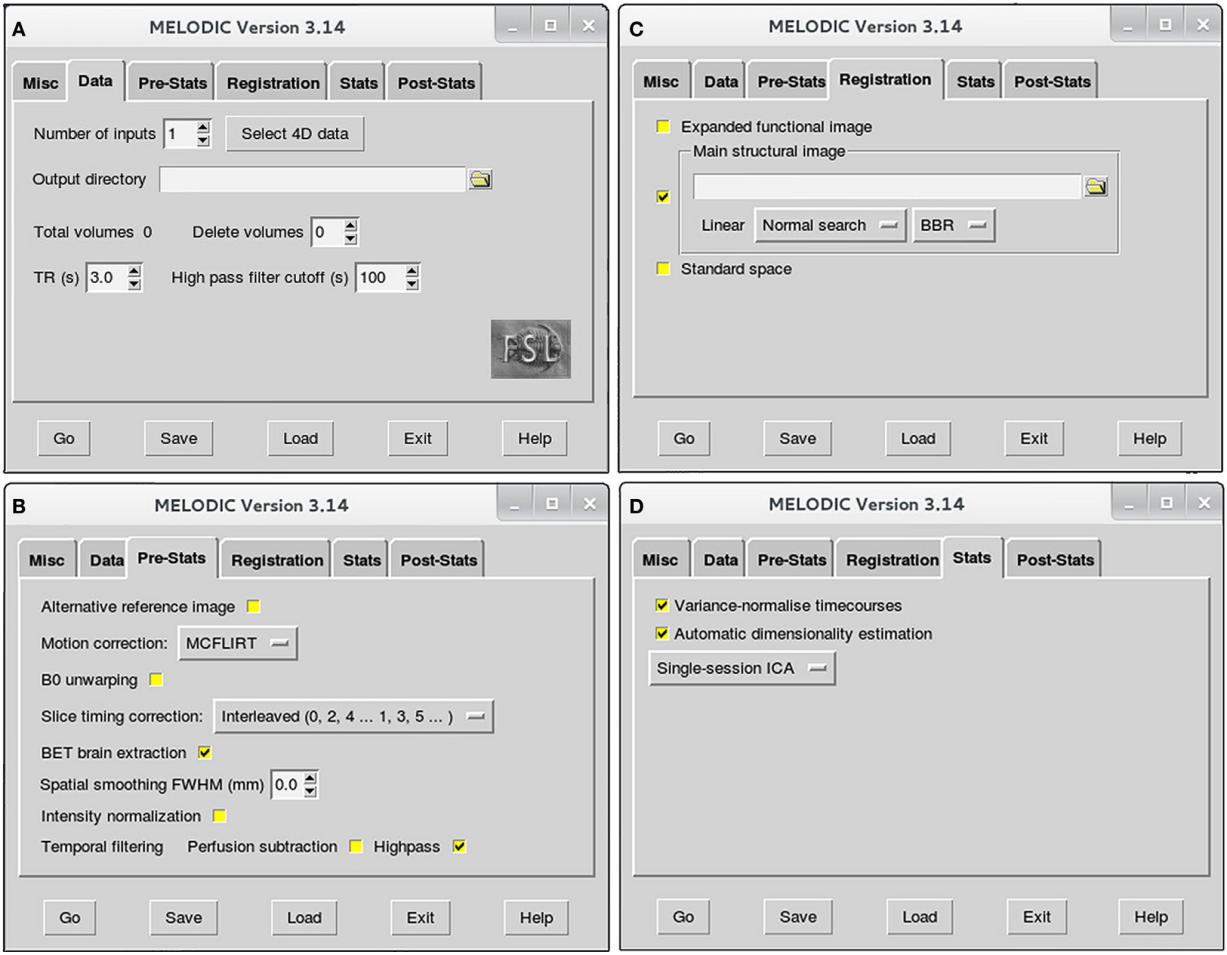
Melodic graphical user interface. Melodic interface is organized into tabs, within which available options for data processing are presented. Selections will be altered in the *Data* **(A)**, *Pre-Stats* **(B)**, *Registration* **(C)**, and *Stats* tabs **(D)** according to the stage of analysis (i.e., preprocessing or final brain network analysis). Melodic interface provides helpful descriptions of each option, viewed by holding ones cursor over a given icon.

To minimize artifact and noise-related signal, many spatial and temporal preprocessing steps are typically performed to prepare raw functional data for statistical analysis. Unique benefits derived from each of these techniques come at the cost of time, on the scale of hours in some cases. Each preprocessing step can be implemented independently, allowing for customization of the preprocessing approach according to individual study design. Currently, there are no established infant-specific parameters for preprocessing infant fMRI data. Parameters used for example infants are provided. However, regardless of subject population, appropriate parameters for any study will ultimately depend on the individual dataset (e.g., length of TR), and cannot simply be adopted from previous literature (51). Offered below is a basic pipeline, with interwoven discussion of the basic theory behind each technique, as well as the rationale behind selecting parameters. All preprocessing steps are implemented simultaneously within Melodic GUI (Figure 2), with the exception of data scrubbing and FIX cleanup (see below, Data Scrubbing and FIX Cleanup, respectively in Section “Preprocessing”). The latter is implemented as part of ICA-based artifact removal—the current recommended 2-step preprocessing approach (44), consisting of PICA followed by FIX (FMRIB’s ICA-based X-noisifier) (60, 61). This approach capitalizes on the ICA model’s strengths, segregating artifactual processes embedded in fMRI data into distinct components, which can then be identified and removed (e.g. by FIX classifier). Within Melodic, pre-statistical preprocessing and registration are carried out using FMRI Expert Analysis Tool (FEAT).

#### Use Melodic for fMRI Data Preprocessing

To open Melodic GUI, type “*Melodic*” into Linux terminal. Once interface opens, instructions to setup Melodic tabs for rs-fMRI data preprocessing are as follows:

➢ *Data* tab (Figure 2A):
  • Number of inputs: select the total number of functional images you wish to analyze at this time, and then input the actual functional files (e.g., *rsnfile*) by pressing “select 4D data.” Indicate where the results of Melodic should be stored by altering “Output directory” name, if applicable.
  • High pass filter cutoff (s): define the desired maximum temporal period for the scan. For the purposes of this protocol, we will set the filter cutoff at 100 s (0.01 Hz). This parameter will eliminate slow temporal drifts (i.e., lower frequencies) whose temporal periods exceed the specified cutoff.
➢ *Pre-Stats* tab (Figure 2B): default settings in this tab will automatically perform grand-mean intensity normalization of the entire 4D dataset by a single multiplicative factor. Additional preprocessing options that require selection include:
  • Motion correction using MCFLIRT^6^: select “MCFLIRT” option from drop down menu during preprocessing run of Melodic. MCFLIRT corrects for head motion during the scan, using the middle volume in time-series as the reference image (62).
  • Slice timing correction: Melodic requires the order in which slices were obtained during scanning to correct for differences in slice timing. As a result, this option will depend on how your fMRI datasets were acquired at the scanner. For example, the current fMRI datasets were acquired in interleaved order, therefore, we change default option “None” to “Interleaved (0, 2, 4…1, 3, 5).” Slice timing correction using Fourier-space time-series phase-shifting improves estimation of functional correlation between voxels in different slices (44). For low-TR data (e.g., subsecond), slice-timing correction may be unnecessary (44).
  • BET brain extraction: select this option during all Melodic runs. This will strip functional images of non-brain tissue, analogous to previous brain extraction of structural image performed in terminal window (see Brain Extraction in Section “Preprocessing”).
  • Spatial smoothing full width at half maximum (FWHM) (mm): turn spatial smoothing off by setting kernel size to 0 mm. Spatial smoothing helps to enhance signal-to-noise ratio (SNR), which describes the degree of distinction between true neuronal signal and artifactual noise. However, it comes at a cost of reduced spatial resolution (63). As a result, spatial smoothing is only used to improve detection of brain activity during final analyses.
  • Temporal filtering: select “Highpass” to remove frequencies from fMRI data whose temporal periods exceed the filter cutoff (specified in *Data* tab). High-pass filtering eliminates linear trends in the data (44), including lower frequencies associated with MRI scanner artifacts (64). Simultaneous low-pass filtering (i.e., bandpass filtering) can be used to eliminate higher frequencies, with oscillatory speeds above a designated threshold. Signal restriction *via* low-pass filtering is not used in this protocol, in accordance with current preprocessing recommendations (44). Though traditionally considered “low-frequency” in resting-state literature, emerging evidence suggests valuable neuronal resting-state signal in infants is present up to and possibly beyond 0.3 Hz (50). Ability to reliably interrogate these higher frequencies is dependent on chosen TR, with longer TRs (e.g., >2 s) resulting in less reliable sampling of BOLD signal fluctuations above 0.17–0.25 Hz (50).
➢ *Registration* tab (Figure 2C): robust linear (affine) registration is carried out using FMRIB’s Linear Image Registration Tool (FLIRT) (62, 65). Each brain-extracted fMRI image (set in *Pre-stats* tab) will be registered to its corresponding structural Reference image (*T1file_ref*). To do this, deselect standardized template option “Standard space” and select “Main structural image.” You will be prompted to select structural images to use for registration. Importantly, structural Reference images must be input in exactly the same order as their corresponding functional files in order to be registered together (specified in *Data* tab; Figure 2A). Verify boundary-based registration (BBR) is selected; this option will enhance the accuracy of gray-matter–white-matter boundary delineations. When projecting functional images into a standard space during GICA, it may be beneficial to implement linear registration for *global* alignment, with a subsequent non-linear registration to enhance *local* alignment using FMRIB’s Non-linear Image Registration Tool (FNIRT; refer to FSL’s website^7^). Non-linear registration typically achieves better alignment for subcortical structures than cortical data (66).
➢ *Stats* tab (Figure 2D): leave all options at the default setting during preprocessing. The following will automatically be selected by Melodic:
  • Variance-normalize timecourses: time-series are rescaled, such that analysis is primarily influenced by voxel-wise temporal dynamics rather than a given voxel’s signal amplitude. More broadly, temporal changes in an area’s signal are used for analysis instead of a given area’s average signal.
  • Automatic dimensionality estimation: this parameter allows for control of the decomposition process of fMRI data into independent components. During preprocessing, the purpose of ICA is to decompose fMRI data into “good” and “bad” components, facilitating subsequent removal of bad components. Selecting “Automatic dimensionality estimation” will instruct probabilistic PCA to automatically estimate the dimensionality (i.e., number of components) of a given functional image, resulting in objective decompositions based on the quality and quantity of data therein (43). Automatic dimensionality estimates should be used during preprocessing, to avoid over- or under-decomposition of the data. This issue will be discussed in more detail during final analysis, at which time a specific dimensionality is typically enforced.
  • Single-session ICA: this option instructs Melodic to analyze individual fMRI data files separately, maintaining session/subject-specific variation. If possible, single-session ICA should always be used for preprocessing stage of analysis, as it improves detection of artifacts that can be highly variable across different scans and/or between subjects.
➢ *Post-stats* tab: leave all options at the default setting during all runs of ICA. The “Threshold IC maps” option is automatically set to 0.5, indicating ICA spatial maps will be thresholded with the alternative hypothesis tested at *P* > 0.5 for activation (signal) versus null (noise).

Once Melodic setup is finished, press “Go” to run the analysis. Computation time for the analysis will scale up with increasing numbers of files included in a given run.

#### Melodic Report

Melodic generates a folder of results for each file run through analysis, in which a Melodic report (report.html) can be found that summarizes results. Once ICA is finished for a given functional file, open its Melodic report and review the results as listed below. This is considered a critical step, as it evaluates motion and registration.

**Figure 3:**
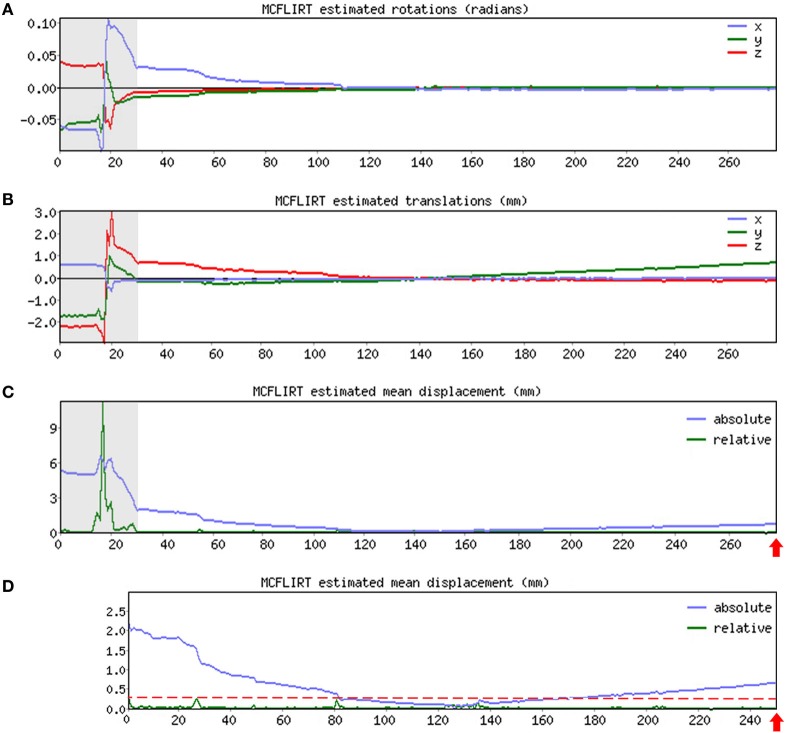
MCFLIRT realignment parameters. Figure illustrates realignment (motion) parameters reported by MCFLIRT for a representative ex-33-week premature infant scanned at 4-weeks corrected age (2.75 months postnatal age). Results are summarized in three graphs, providing an index of head position throughout the scan for each volume in the time-series (*x*-axis; 280 total volumes). Estimated rotation (in radians; A) and translation (in millimeters; B) parameters are described with respect to each axis (*x, y, z*). In panel **(C)**, aforementioned parameters are condensed by MCFLIRT into a single vector referred to as root mean squared (RMS) displacement (in millimeters), which summarizes cumulative motion in terms of absolute and relative measures. Absolute RMS displacement (blue) describes motion in a given volume with respect to a reference time point (i.e., middle volume in time-series), providing useful information on gradual shifts in head position over time. Relative RMS displacement (green) at a given volume describes motion with respect to the subsequent time point, useful for identifying abrupt changes in head position. Gray regions highlight initial 28 volumes in time-series that contain excessive motion (e.g., spikes >2.5 mm *absolute* or >0.25 mm *relative* displacements), surpassing established parameter criteria for inclusion. Importantly, gray region extends beyond obvious jump in relative displacement to include steep declining slope in absolute displacement. This is to address residual effects of motion that often extend beyond apparent spikes in relative displacement (51). These volumes are removed via data scrubbing (see Data Scrubbing in Section “Preprocessing”). Panel **(D)** shows RMS displacement (cumulative motion) realignment parameters after data scrubbing. Reduction in total number of volumes from 280 to 252 (red arrows) reflects removal of initial 28 volumes (gray regions removed). Note attenuated motion parameters (*y*-axis; green) throughout scan. Importantly, relative displacement, remains below threshold criteria for duration of scan (red dashed line). Mean RMS displacements of original time-series (*absolute*, 1.05 mm; *relative*, 0.13 mm) are greatly reduced by data scrubbing (*absolute*, 0.58 mm; *relative*, 0.03 mm).

**Figure 4:**
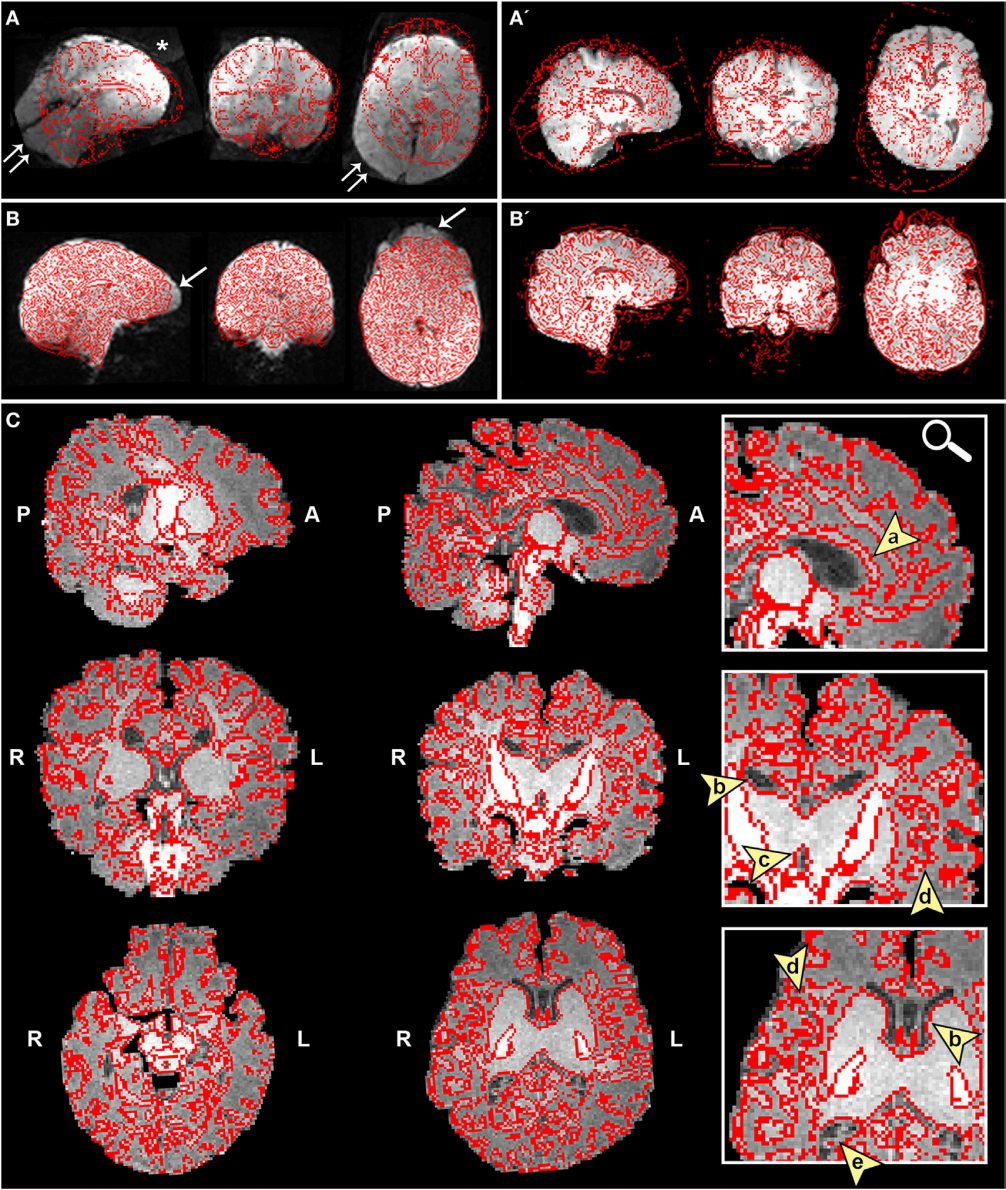
Registration of functional magnetic resonance imaging (fMRI) image to native space. Figure illustrates examples of poor **(A and A′)** and reasonable **(B and B′)** registration of fMRI data to native space as they appear in the Melodic report, using representative ex-33-week premature infant scanned at 4-weeks corrected age (2.75 months postnatal age) and full-term infant scanned at 5 months of age, respectively. Each row shows structural-to-functional image alignment in parasagittal, coronal, and axial views (from left to right). In panels **(A)** and **(B)**, fMRI image is shown in gray-scale, while overlying red contours delineate corresponding structural Reference image (also referred to as infant’s native space). In panels (A′) and **(B′)**, this relationship is reversed (i.e., red contour represents functional image). Panel **(A)** illustrates poor functional-to-structural image alignment, likely precipitated in part by magnetic field inhomogeneity (i.e., susceptibility effects). The latter causes apparent EPI-related distortions (double arrows), negatively influencing registration. Misalignment presents as improper/absent contours delineating gray and white matter boundaries, ventricles, and outline of the brain (best viewed in coronal sections). Note the common echo-planar imaging (EPI) sequence artifact referred to as “ghosting” (white asterisk). In panel **(B)**, registration results demonstrate reasonable alignment, with minor distortion artifact noted in the orbitofrontal region (single arrows) that are commonly observed in regions adjacent to air-tissue interfaces like the nasal cavity and sinuses (72). For coronal and transverse sections, left hemisphere of the brain corresponds to the right side of the image. Panel **(C)** illustrates an alternative method for visualizing registration results post-independent component analysis using representative ex-25-week premature infant scanned at term-equivalent age (4.25 months postnatal age; see also Figure 8). White matter contours of functional data are shown in red overlaid on high-resolution structural T1 image, with arrowheads highlighting good structural-to-functional overlap of tissue boundaries (a, corpus callosum; b, body of lateral ventricles; c, third ventricle; d, insular cortex; e, occipital horns of lateral ventricles). Abbreviations: A, anterior; L, left; P, posterior; R, right.

➢ Pre-stats section: MCFLIRT realignment parameters. Confirm the presence or absence of excessive motion in time-series by reviewing the results of MCFLIRT (Figures 3A–C). As previously stated, an appropriate definition of excessive motion will depend entirely upon the individual dataset (e.g., length of TR), and cannot simply be adopted from previous literature (51). Threshold criteria defined in this protocol include root mean squared (RMS) *relative* displacement >0.25 mm or RMS *absolute* displacement >2.5 mm, as well as translational motion exceeding voxel size (e.g., >2 mm). An additional measure of motion gaining in popularity called derivative of RMS variance over voxels (DVARS) describes changes in signal intensity from volume-to-volume that strongly correlate with relative RMS displacement (51). If definition of excessive motion is met, assess whether data scrubbing (i.e., targeted volume removal) can be used to salvage the time-series (see Data Scrubbing in Section “Preprocessing”).
➢ Registration section. Ensure proper alignment of functional and structural images (Figure 4). If registration is suboptimal, several avenues exist to fix mis-registration. One option would be to perform BET on the functional image output by Melodic called *example_func*, followed by bias field correction using FAST to normalize signal intensities (see Bias Field Correction in Section “Preprocessing”). Alternatively, one can rerun FLIRT using the *example_func* file as the initial functional image during registration. Poor registration may also result from magnetic field inhomogeneity, presenting as EPI distortions (e.g., stretching or warping) in the fMRI image (Figure 4B). Multiple correction methods exist to undo susceptibility-induced distortion, including use of “top–down” distortion correction (67), “top-up” distortion correction (44, 66, 68), a self-field map (69, 70), or a mean field map (71). The latter three options necessitate pre-planning, requiring an additional scan during initial data acquisition phase.
➢ ICA section. As previously mentioned, PCA is allowed to objectively estimate the dimensionality of each subject’s fMRI data during this initial run of ICA. As a result, the number of components extracted will vary between subjects/scans. This section does not require immediate actions at this time, however, these preliminary ICA maps will be referenced later in the text during supervision of FIX denoising (see FIX Cleanup in Section “Preprocessing”).

##### Important Point of Functional Preprocessing

Both excessive motion and poor registration will negatively impact the accuracy of subsequent analyses, negating significance of any findings thereafter. If either issue cannot be rectified, the general consensus is that offending data should be altogether eliminated from further analyses.

#### Data Scrubbing

As illustrated in Figure 3, if definition of excessive motion was met during review of Melodic report (see Melodic Report in Section “Preprocessing”), data scrubbing (i.e., volume censoring) is an effective strategy to reduce the influence of motion-afflicted volumes in later analyses. Specifically, motion-corrupted time-points can be excised from rs-fMRI time-series, with two caveats: the time-series must retain temporal contiguity (i.e., disparate lengths of volumes cannot be re-combined), while still complying with chosen time-series length criterion (see Time-Series Length Criterion in Section “Data Quality Assessment”). This means that time-series containing centrally located motion-corrupted volumes are typically unsalvageable. However, motion-corrupted volumes located near the beginning or end of time-series can typically be removed by isolating the region of interest (i.e., volumes without motion; Figures 3A–C). To isolate this length of time-series, use command: *fslroi* <rsnfile> <rsnfile_rdcd> <#1> <#2> (“roi,” region of interest; “_rdcd,” output file with reduced time-series). First value <#1> indicates the new starting volume at which the newly reduced time-series will begin. Second value listed <#2> indicates the number volumes to be included following new designated starting volume (i.e., the length of the new time-series). Output file (*rsnfile_rdcd*) will automatically save in the same directory as the input file containing original fMRI time-series (*rsnfile*). An illustration of data scrubbing to reduce motion contamination is shown in Figure 3D. Several notes should be addressed and include:

- Note on volume numbering. In the Melodic report, the first volume in a given time-series corresponds to “volume 1” (e.g., MCFLIRT graphs), however, FSL defines the first volume as “volume zero.” When using the *fslroi* command to remove volumes, verify the two values designated in command are in line with FSL’s definition.
- Note on data scrubbing. For some processing streams (e.g., multivariate pattern analysis), it may be necessary to reduce all time-series to equal lengths in order to retain equal degrees of freedom across subjects (51). If data scrubbing is performed, the new file containing reduced time-series must undergo all fMRI data preprocessing steps from the beginning (i.e., Use Melodic for fMRI Data Preprocessing and Melodic Report in Section “Preprocessing”). Preprocessing steps will be identical for newly scrubbed file, with one exception. In the *Data* tab, input functional file containing the newly reduced BOLD time-series (e.g., *rsnfile_rdcd*), rather than selecting the original fMRI data (e.g., *rsnfile*). Once preprocessing rerun of Melodic is finished, one should review the Melodic report to verify motion-corrupted volumes were successfully removed, and motion criterion is met.

#### FIX Cleanup

Denoising of fMRI data can be achieved using FMRIB’s ICA-based X-noiseifier (FIX) hierarchical classifier, implemented within FSL (60, 61). FIX is an ensemble learner^8^ (i.e., fusion classifier) designed to address the complexity of component (i.e., network) classification created by signal heterogeneity, often referred to as component “impurity” (i.e., components realistically contain a mixture of both signal and noise) (61). Accordingly, removal of any one component engenders risk of eliminating valuable neuronal signal. Collectively, FIX analyses each ICA component for over 180 spatial and temporal features (61), ultimately culminating in a final weighted prediction. Specifically, components are classified as “good” (i.e., predominately brain activity), “bad” (i.e., predominantly artifact), or “unknown” signal origins. Only the unique variance associated with “bad” components, reflecting predominance of non-neuronal physiological signals and/or motion-related timecourses, is eliminated to create “cleaned” 4D fMRI datasets (44, 73). To run FIX cleanup, use the following command: *fix* <mel.ica> <train>.RData <thr>.

Here, <mel.ica> represents the output folder from preprocessing Melodic run, containing all files necessary for FIX analysis (e.g., preprocessed fMRI data, motion parameters, structural Reference image). Option <train> is the full file directory leading to location of training weights, which guide FIX classifications (discussed below). The threshold parameter <thr> ranges from 0 to 100, but should realistically be limited to 5–20. Given that FIX output is a probability, threshold parameter controls the balance between FIX’s two accuracies: (1) correct identification of good components (i.e., true-positive-rate), and (2) correct identification of bad components (i.e., true-negative-rate). Low thresholds (e.g., 5) favor true-positive-rate, resulting in a conservative cleanup. As the threshold parameter is increased, FIX emphasis on true-positive-rate shifts increasingly in favor of true-negative-rate (at the expense of true-positive-rate). Optimal threshold will vary depending on which of these accuracies is prioritized in ones analysis. Typically, the strategy for ICA-based cleaning methods holds to the principle “innocent until proven guilty,” where only components clearly from artifactual origins are eliminated during preprocessing (true-positive-rate >true-negative-rate) (74). Past investigations in adults using threshold parameters 5–10 found good (>95%) to excellent (>99%) mean accuracy of FIX component classifications compared to manual operators, as well as a good balance between ratio of true-positive-rate/true-negative-rate (61). Once finished, FIX generates two output files of interest: (1) a text file listing all component classifications, including the “bad” components removed, and (2) a file containing new “cleaned” version of fMRI data, which is used to perform final statistical analysis.

##### Notes on Employing FIX Cleanup

Preexisting training weight files are supplied with FIX and can be used provided scan acquisition parameters are similar. Infant data presented herein used FIX guided by preexisting Human Connectome Project training files, intended for use on minimally processed adult datasets (e.g., no spatial smoothing) acquired with similar parameters as Human Connectome Project fMRI data (44, 61). In these instances, a more conservative threshold should be used to mitigate loss of valuable signal, given age-specific physiological parameters (see Frequency Characteristics in Section “Discussion”). Our work employs a threshold of 10. Study-specific training datasets are highly recommended to improve FIX classification accuracies, provided there are a sufficient number of training datasets (>10 subjects) to do so (61). Recently, Ball et al. (75) were the first ever to apply FIX classifier (trained on 40 preterm infant datasets) on infant rs-fMRI data, and reported highest FIX accuracies using a threshold of 20. Additional factors such as subject number and extent of motion were also shown to correlate with relative accuracies of FIX (75). Datasets used to train FIX should be distinct from infant data intended for analysis, or one risks biasing the classifier. For smaller sample sizes or unique patient populations, it is advisable to manually classify (by hand) all components extracted by ICA. In these cases, non-automated denoising of fMRI data can be achieved using the command *fsl_regfilt* within terminal window. This command allows one to list components to be removed from the data (analogous to FIX). Refer to the FSL website for information regarding available training weights, as well as detailed instructions on how to re-train FIX^9^ and how to manually denoise fMRI data.^10^ It is important to monitor the performance of FIX for consistency and accuracy. Recording the % of total ICA components removed by FIX from each subject’s fMRI data is one avenue to monitor FIX’s consistency at a given threshold. One should expect a high proportion of noise components identified in the preprocessing run of ICA. Past investigations, using data acquired with both standard and multiband sequences on 3T scanners, consistently reported a predominance of noise components on the order of 70–90% (44, 60, 76, 77). These reports highlight the incentive of using ICA-based artifact removal. It will also be crucial to monitor the accuracy of FIX classifications, particularly if the classifier is not trained on study-specific fMRI datasets (61). This can be achieved by cross-examining component classifications made by FIX with component features recorded in the Melodic report (e.g., spatial map, timecourse, powerspectra). This is especially important for components that were removed by FIX from fMRI data in order to preserve as much neural signal as possible. Melodic components are listed in order of explained variance (i.e., contributors of noise), such that components containing the highest % of explained variance appear first and typically contain little to no valuable signal (74). Accordingly, one should expect to see a greater number of components listed toward the beginning of Melodic report to appear among the components identified as artifact and removed by FIX. A general guide to assess ICA component spatiotemporal signal characteristics is described in the next section (see ICA Results: Evaluation and Data Presentation in Section “Final Analysis”).

## Method Part III. Final Analysis

Final statistical analysis resulting in extraction of RSNs will entail (1) a second run of single-session ICA or GICA using cleaned fMRI data, followed by (2) evaluation of resultant independent components to identify large-scale neural networks.

### Final Statistical Analysis Using ICA

Final analysis is performed using ICA, implemented again within the Melodic interface. This final run is used to measure temporal coherence of brain activity between different brain regions (i.e., functional connectivity), resulting in extraction of resting-state brain networks, as well as some artifactual components. Setup for *Registration* and *Post-Stats* tabs are identical to the procedure outlined for previous Melodic run (see Use Melodic for fMRI Data Preprocessing in Section “Preprocessing”). Tab selections unique to final analysis are noted below, as well as distinguishing options to perform GICA:

➢ *Data* tab (Figure 2A): follow instructions described in previous Melodic run, except for “Select 4D data,” where one should input cleaned version fMRI data. Numerous files can be input at one time and the type of analysis (see *Stats* Tab below) will determine how each file is analyzed: individually (*viz*. single-session ICA) or as a group (*viz*. GICA).
➢ *Pre-stats* tab (Figure 2B): deselect “Highpass” temporal filtering, and select “None” for motion correction and slice timing correction. These steps were already performed during preprocessing stage. Pre-statistical processing steps needed during final analysis are as follows:
  • BET brain extraction: select this option.
  • Spatial smoothing FWHM^11^ (mm): select a Gaussian kernel size (mm) to apply spatial smoothing. Spatial smoothing is used during final network analysis to improve SNR and reduce minor registration imperfections, greatly improving accurate detection of true neuronal signal (63). Spatial smoothing itself is achieved by applying a Gaussian kernel size (mm). Optimal kernel size will depend on brain size (e.g., neonate versus toddler), the quality of data (e.g., SNR), as well as the size of the brain activity ROI. Larger sizes are useful in instances of poor SNR and when patterns of brain activity are expected to cover large regions. For the purpose of infant brain analysis, we have chosen a kernel size of 5 mm.
➢ *Registration* tab (Figure 2C): if using single-session ICA for final analysis, each infant’s fMRI image is registered to its native space (e.g., T1 Reference image), and tab setup is identical to previous Melodic run (see Use Melodic for fMRI Data Preprocessing in Section “Preprocessing”). If using multi-subject GICA, each subject’s fMRI data must be transformed into a standardized coordinate space. Accordingly, one should select option “Standard space” and designate a standardized anatomical template or atlas. Infant-specific challenges associated with GICA registration, including lack of standardized age-specific atlases, will be discussed at length in the *Discussion* section (see Age-Specific Atlases in Section “Methodological Challenges”).
➢ *Stats* tab (Figure 2D):
  • Variance-normalize timecourses: leave in default settings to rescale time-series, same as prior Melodic run (see Use Melodic for fMRI Data Preprocessing in Section “Preprocessing”).
  • Automatic dimensionality estimation: during final analysis, one may choose to enforce a uniform dimensionality for all subjects. Deselect “automatic dimensionality estimation” and designate the desired dimensionality. Currently, there is no consensus on how best to estimate optimal dimensionality of a given dataset. Recent evidence suggests a range of dimensionalities may be used to extract interpretable networks (22). Speculatively, “splitting” of lower-dimensional networks into sub-networks (or network nodes) at higher dimensionalities is thought to reflect functional hierarchy (11, 22, 44). At higher dimensionalities, components tend to be more functionally homogeneous (desirable), but exhibit noisier associated timecourses (undesirable), as fewer time-series are averaged together (44). Further, while higher dimensionalities arguably provide more biological detail, too high a decomposition may compromise attempts at comparative analysis due to subject spatial variability (e.g., the probability that Subject A and Subject B share the same functional connectivity at a given brain ROI will decrease as the ROI dwindles in size) (44). Ultimately, optimal dimensionality will depend on the intent of analysis, as well as the quality and quantity of fMRI data (44). For datasets of representative infants in the current protocol, we enforced a dimensionality of 40. This produced reasonable decomposition of fMRI data into interpretable networks and sub-components, achieving a balance between component convergence and splitting.
  • Single-session ICA: during final analysis, one can use either single-session ICA to retain session/subject-specific variation, or select “multi-session temporal concatenation” from the drop-down menu to perform GICA and retain variation representative of the whole sample. GICA uses multi-subject datasets concatenated into a single lengthy time-series, which is then analyzed by ICA to produce group-average spatial maps. Group analysis has been suggested to provide robust, detailed functional decompositions more reliably than single-subject analyses (44). However, it is not without its downsides. For instance, group-wise comparison necessitates arbitrary selection of appropriate age ranges, for which there is currently no consensus. During the postnatal period, significant changes in structural and functional architecture are known to occur on the order of weeks (78–80). As such, rapidity of brain development may confound efforts to perform group-level analyses if age parameters are inappropriate (e.g., too broad; see Age-Specific Atlases in Section “Discussion”).

Once Melodic setup is finished, press “Go” to run the final analysis. The Melodic output file containing extracted components will be named *melodic_IC*.

### Dual Regression (Only for GICA)

Group-level analysis can identify large-scale patterns of functional connectivity in a given sample, effectively defining functional networks of interest particularly useful for group comparisons. As noted, GICA generates group-level spatial maps that reflect the average functional connectivity across all subjects in that group. An additional step (e.g., dual regression) is needed to estimate individual subject spatial maps from the group-average. Dual regression is a two-stage process (i.e., multiple linear regression) used to identify spatial maps and associated timecourses for individual fMRI images that correspond to those networks derived from group-level analysis (81). This approach probes between-subject group-consistency in network connectivity, allowing for identification of between-subject group-differences with high accuracy compared to back-projection methods, which can produce false statistical significance (i.e., false-positives or false-negatives) (81). For instructions on creating meaningful multi-subject experimental designs, refer to the FSL website.^12^ Dual regression results in subject-specific timecourses and spatial maps corresponding to group-level components, as well as tstat images that correspond to group contrasts (e.g., main effects and interactions) in the chosen design matrix. To fully appreciate the value of dual regression outputs and their implications in the context of a study, one should refer to the published literature [e.g., Ref. (82–84)].

### ICA Results: Evaluation and Data Presentation

Probabilistic ICA extracts a given number of statistically independent components, segregated during data decomposition based on temporal covariance of BOLD signal. Accordingly, signal arising from artifactual sources (e.g., cardiac pulsation) helpfully groups together, predominantly isolated from true neuronal signal reflecting functional brain networks. However, the ICA algorithm does not specify component classifications, necessitating manual identification. Accordingly, each individual component must be inspected one-by-one during preprocessing, for accurate denoising by FIX, as well as following final statistical analysis to identify finalized brain networks. At both stages, it is normal to see a large number of noise components in the ICA results. Described below are common spatiotemporal component characteristics used to inform classifications, as well as visualization strategies for efficient evaluation of component features.

#### How to Visualize Components

Effective viewers are available that allow for simultaneous visualization of spatial, temporal, and spectral features. Freely available programs include:

- *Melview*,^13^ embedded within FSLeyes (replacement for FSLView in latest version of FSL^14^)
- *Connectome Workbench* used to evaluate data from the Human Connectome Project^15^
- Analysis of Functional Neuroimages (AFNI)^16^
- GICA of fMRI Toolbox (GIFT; see text footnote 5).

**Figure 5:**
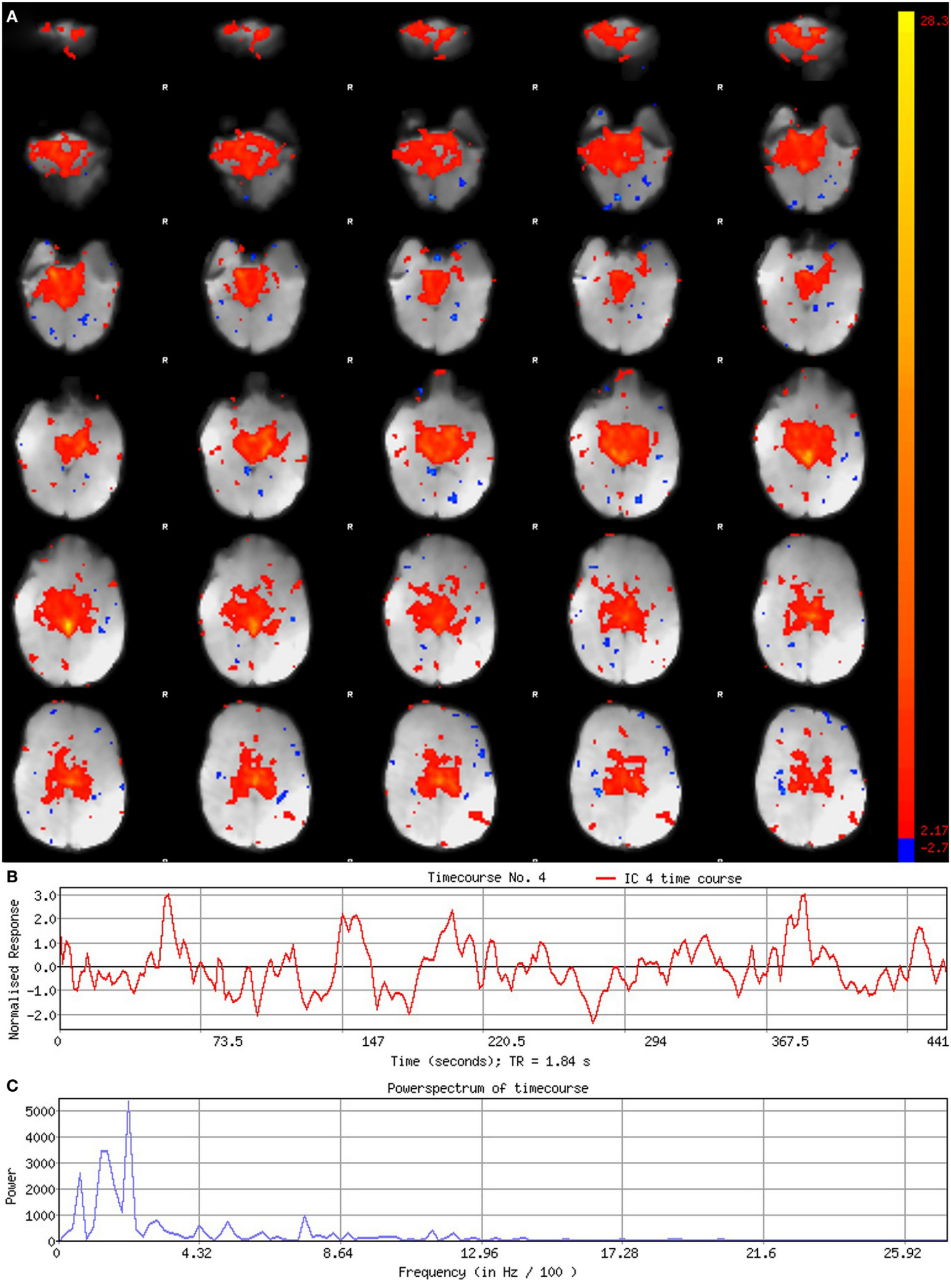
Signal component. Figure illustrates representative signal component obtained from final analysis of an ex-33-week premature infant scanned at 4-weeks corrected age (2.75 months postnatal age). Panel **(A)** shows component’s spatial map, presented in the Melodic report as *z*-scores superimposed on serial axial sections of mean functional image in radiological convention (right side of image corresponds to left side of brain). Its spatial distribution demonstrates gradual change through progressive sections, encompassing biologically relevant structures consistent with the cerebellar-subcortical network (also shown in Figure 8). In panel **(B)**, the component’s associated timecourse follows a regular, oscillatory pattern, with no sudden spikes. The power spectrum in panel **(C)** shows a predominance of high power at low frequencies, characteristic of resting-state neural networks.

**Figure 6:**
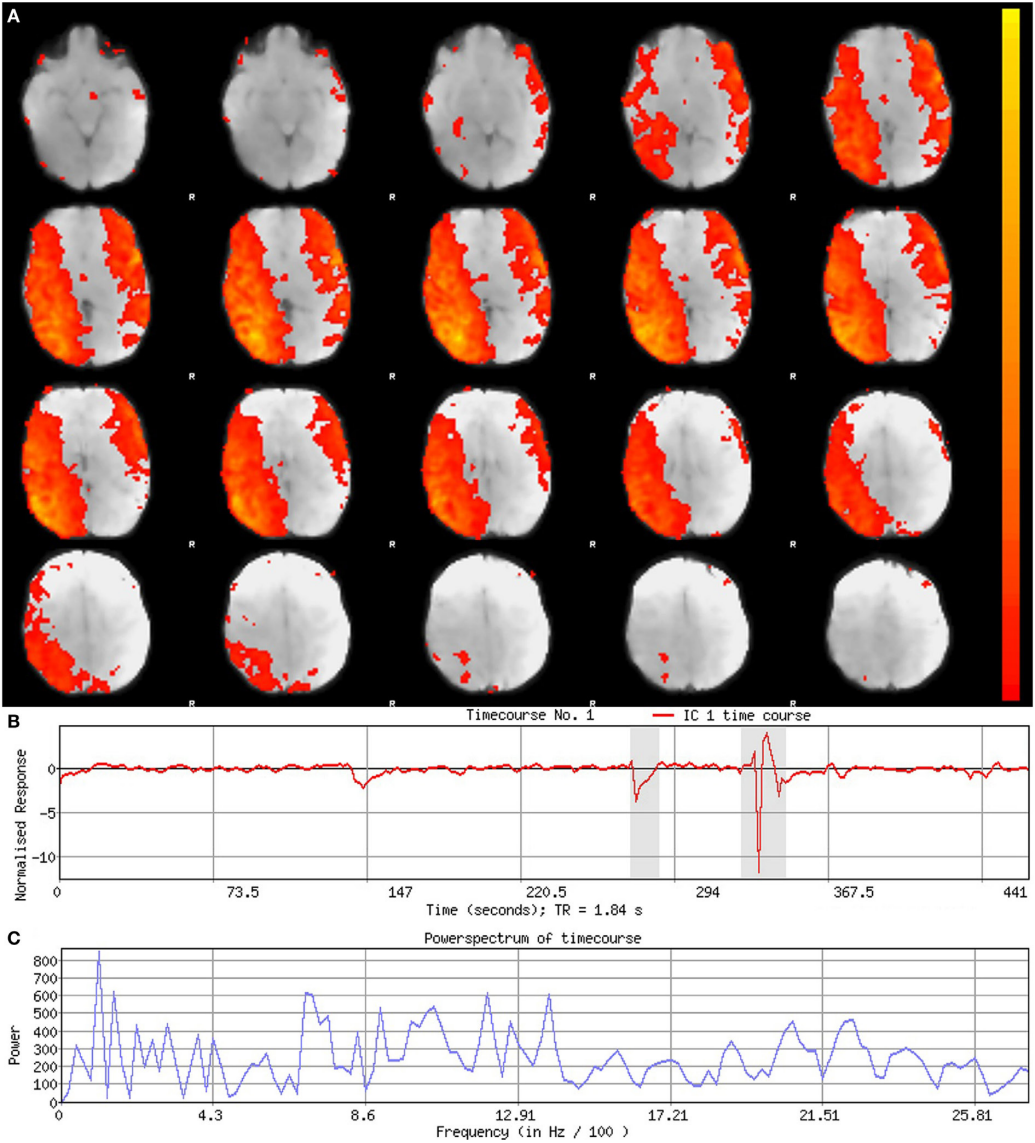
Noise component. Figure illustrates representative noise component in Melodic report obtained from final analysis of an ex-33-week premature infant scanned at 9-weeks corrected age (4 months postnatal age). In panel **(A)**, component’s spatial map is presented as *z*-scores, superimposed on mean functional image in radiological convention (right side of image corresponds to left side of brain). Component characteristics are indicative of artifact, demonstrating diffuse and anatomically inconsistent spatial activations **(A)**, sudden jumps (highlighted in gray) in associated timecourse **(B)**, as well as pan frequency distribution of spectral power [i.e., persistent spectral power across entire frequency range **(C)**].

**Figure 7:**
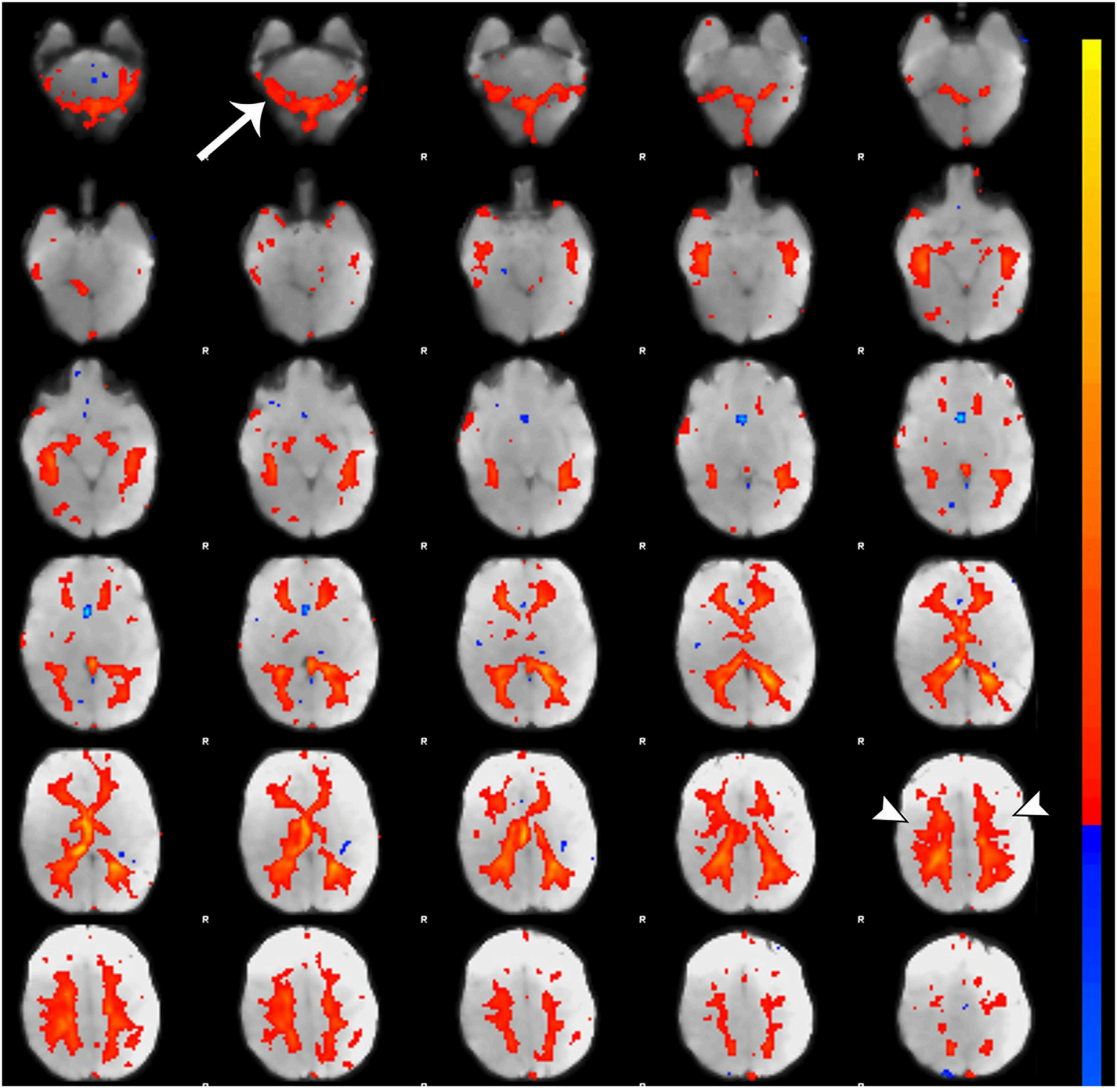
Noise component related to subependymal and transmedullary veins. Figure illustrates representative noise component in Melodic report obtained from final analysis of an ex-33-week premature infant scanned at 9-weeks corrected age (4 months postnatal age). X-shaped spatial distribution generated by pulsation in subependymal and to a lesser degree transmedullary veins (85, 86). These components are often mistaken for white matter components (arrowheads) due to the effect of spatial smoothing on signal distribution (74). Note the presence of rim-like activity in lower slices near the cerebellum (single arrow), which is a common finding in noise components. Melodic report spatial map is presented as *z*-scores superimposed on mean functional image in radiological convention (right side of image corresponds to left side of brain).

**Figure 8:**
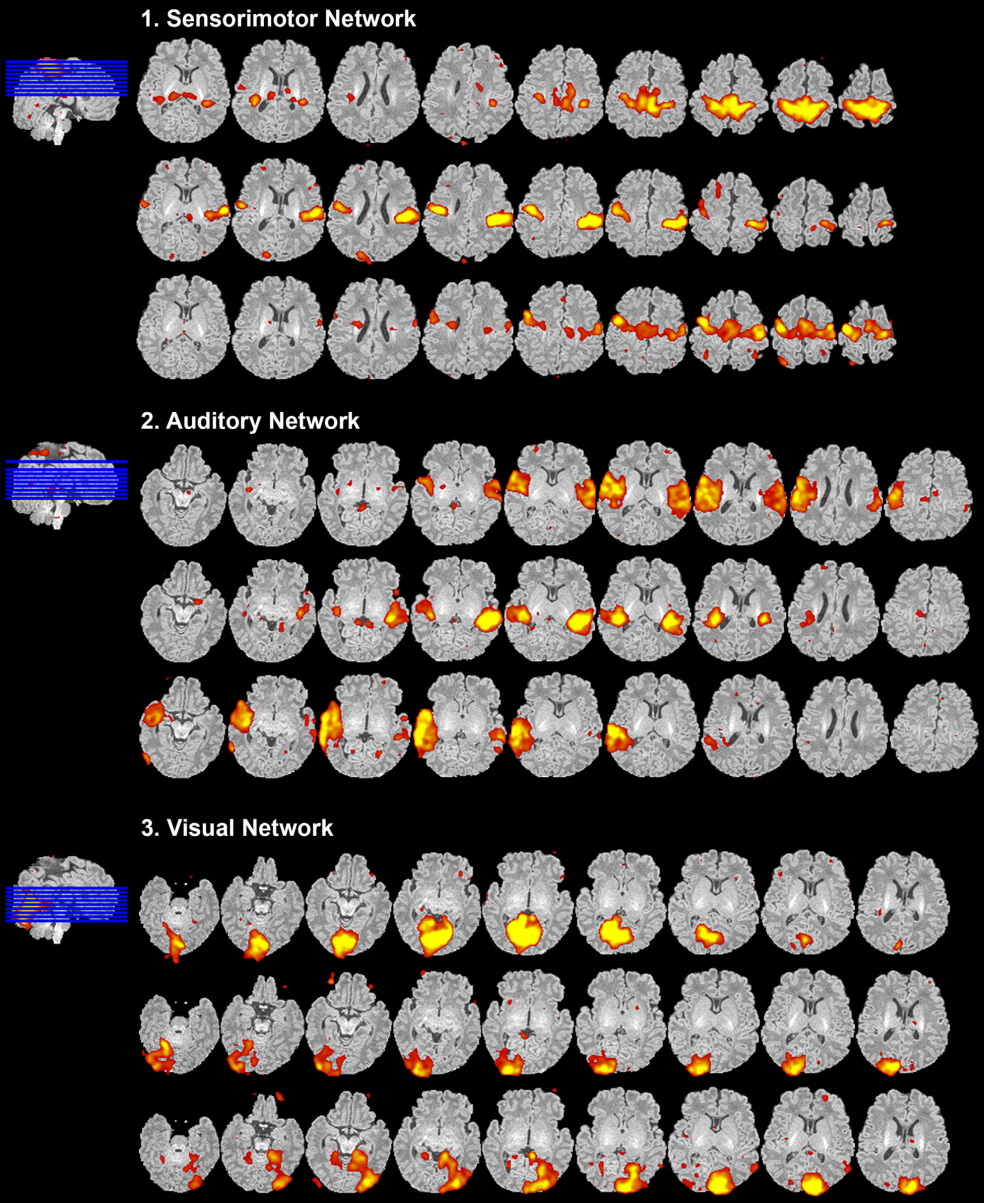

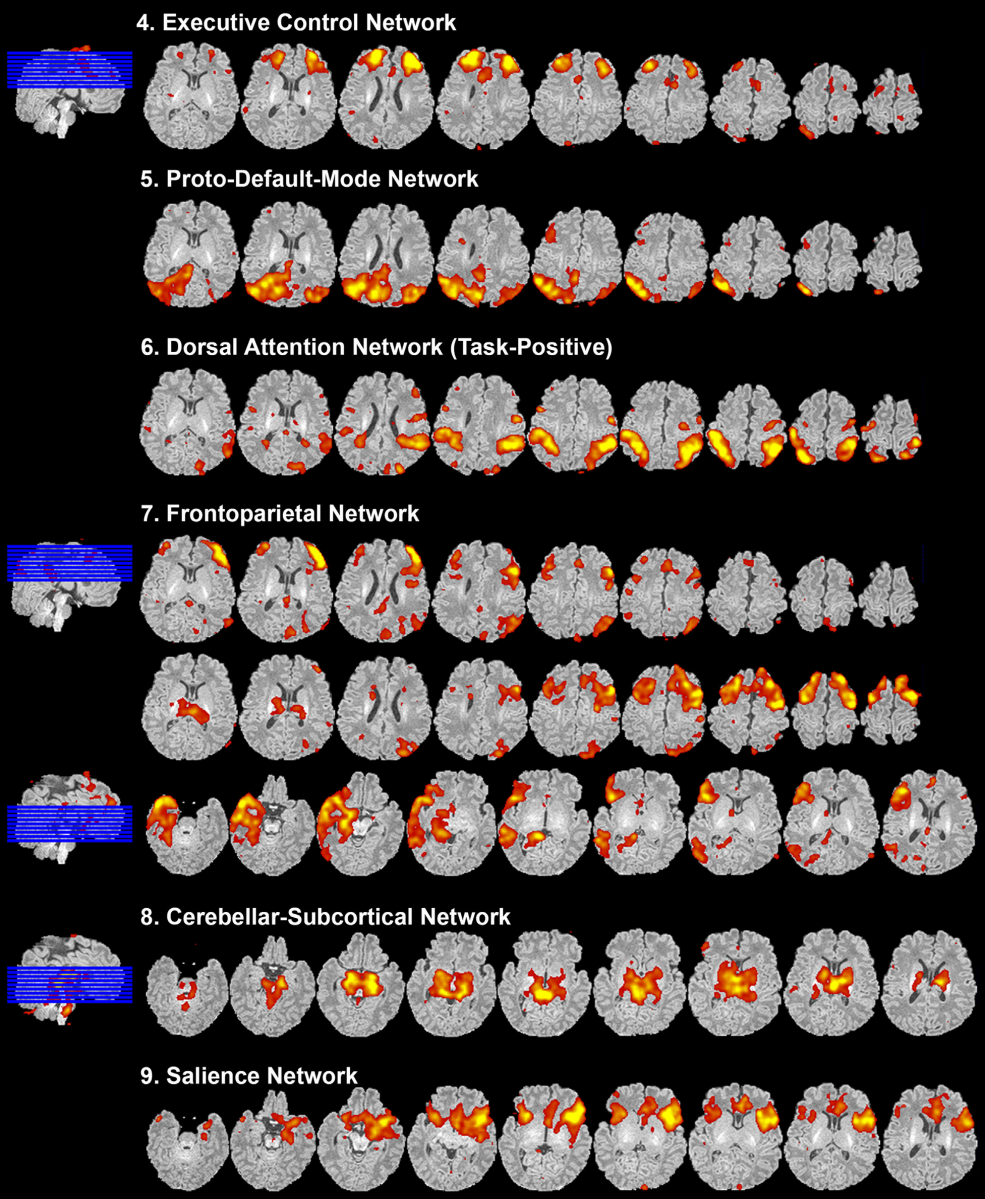
Resting-state network data presentation. Figure illustrates representative neural components (18 out of 40) comprising eight total resting-state networks obtained from final analysis of an ex-25-week premature infant scanned at term-equivalent age (4.25 months postnatal age). Identified networks were as follows: (1) sensorimotor, (2) auditory, (3) visual, (4) executive control, (5) proto-default-mode, (6) dorsal attention, (7) frontoparietal, (8) cerebellar-subcortical, and (9) salience networks. Identified networks were spatially consistent with those previously described in infants (22, 25–29, 32, 33, 87–89) and adults (11, 90, 91). Spatial maps are presented as *z*-scores (arbitrarily thresholded at *z* = 2.8) superimposed on corresponding structural Reference image, and presented in serial axial view using MRIcron. Slices progress from ventral to more dorsal sections (left to right). In accordance with traditional radiological convention, right side of image corresponds to left side of brain.

Each program offers unique benefits for efficient evaluation of components. Components are usually displayed overlaid on associated mean EPI image (e.g., in the Melodic report, Figures 5–7). Current methodological challenges are associated with acquisition (e.g., limited gradient capabilities) that limits voxel size of fMRI images to 2 mm × 2 mm × 2 mm or larger (21). While this may be sufficient in adults, constrained voxel size results in poor spatial resolution in infant functional MR images due to smaller brain size (21). To combat this during component evaluations, it is generally advisable to overlay components (e.g., *melodic_IC*) on corresponding high-resolution structural images (Figure 8), which can be achieved within FSLview or MRIcron,^17^ and scroll through sections in each orthogonal plane. MRIcron provides a user-friendly way to generate serial axial sections of each component, helpful for identifying networks and facilitating intra- and intersubject comparisons. This is clearly illustrated in Figure 8, where a representative infant’s fMRI image was aligned to its native space (i.e., T1 Reference image), rather than projected into a shared standard space. Ultimately, the utility of overlaying components on structural images is entirely dependent on proper structural-functional image alignment (see Figure 4).

#### Component Characteristics

Qualitative evaluation of an individual component to determine whether it reflects neuronal or artifactual signal can be obvious or more obscure. Spatial map distribution (spatial features), associated timecourses (temporal features), and powerspectra (spectral features) of each component should all factor in to final classification (Figures 5–7). Additionally, comparison of timecourses with MCFLIRT realignment parameters obtained during preprocessing (see Figure 3) can reveal any corresponding spikes between the two time-series, indicative of motion artifact. Refer to recently published work by Griffanti et al. (74) for an in depth “how-to” guide on component classification, including extensive illustrations of component characteristics, as well as a hierarchical flow-chart of the decision-making process. A brief review of typical features associated with signal and noise components are described below in hierarchical order of importance.

##### Spatial Features

Spatial maps of neuronal components have patchy, “area-like” distributions localized within gray matter tissue, typically involving both gyri and sulci (44).^18^ Primordial RSNs in infants before or around term-equivalent age are often described as “local blooms” (i.e., network spatial diffusivity), whereas older infants tend to exhibit more localized signal (33). Signal in subcortical brain regions should also be localized to gray matter (e.g., Figures 5A and 8). Noise components may exhibit banding patterns, as well as signal distributions inconsistent with anatomical boundaries (e.g., Figure 6A) or localized within areas of susceptibility-induced signal loss (e.g., orbitofrontal regions) are likely artifact. Furthermore, component maps showing spatial overlap predominantly in areas of white matter, CSF (e.g., cisterns or ventricles), venous sinuses, or cerebral vasculature (e.g., subependymal veins; Figure 7) are likely artifact. Signal concentrated on gyral crowns (without sulci involvement) that presents as “arc-like” spatial distributions are artifact and may be motion-related (not shown) (44). Importantly, lateralized components may be a reflection of: normal [e.g., lateralization of language (92)] or abnormal, [e.g., prematurity (93) or stress (94)] network development; inappropriate dimensionality parameter (i.e., overfitting); or the manifestation of residual motion in the data. Complementary lateralized components for a given network may be the result of “splitting” due to inappropriately high dimensionality (see Final Statistical Analysis Using ICA in Section “Final Analysis”). Due to the potentially multifactorial etiology of lateralization, such components should be interpreted with the upmost caution.

##### Temporal Features

Timecourses of neuronal components follow a relatively regular oscillatory path (distinct from physiological oscillation patterns), with no sudden jumps (e.g., Figure 5B). Temporal features including spikes in time-series (motion; Figure 6B), or oscillatory patterns (physiologic noise) indicate presence of artifact.

##### Spectral Features

Spectral features of RSNs typically show power distribution concentrated at lower frequencies (0.01–0.1 Hz), with at least one strong peak therein (e.g., Figure 5C) (44). Power spectra contaminated by non-neuronal noise will often show an abundance of high or very low frequencies inconsistent with characteristic range of low frequency RSNs as well as pan frequency distributions (74). The latter is illustrated in Figure 6C.

#### Correlation Thresholds

For each component, *z*-score maps should be inspected in both the raw format, and after application of a default threshold (typically around 2–3). Higher thresholds applied to spatial maps are helpful for localizing signal to identify regions of strongest correlation (e.g., bright yellow), as well as generating unsmoothed component maps. Lowering threshold can also be used to discern the source of signal in smaller, weaker correlated regions. For example, small patches of signal from neuronal sources tend to gradually expand, spatially aligned with gyral convolutions. In contrast, signal stemming from artifactual sources may remain small, stagnant entities or expand, but with disregard for contours of brain tissue (74). Threshold parameters specific to each component can be found by looking at the color bar next to its spatial map within the Melodic report (shown in Figures 5–7). The color bar provides an index of correlational strength, with warm red–yellow colors reflecting positive correlations and blue–green cool colors denoting negative correlations. Raw *z*-score maps may show negative and/or positive clusters. A predominance of negative correlations does not necessarily condemn a component as artifactual noise. Anticorrelated RSNs in adults are thought to reflect intrinsically anticorrelated functional systems (95). An example of this is a “task-positive” system comprised of brain regions that routinely exhibit increased activity during attention demanding tasks, and corresponding “task-negative” system that shows the opposite (95).

#### Presentation of Results

After carefully reviewing all ICA components (both signal and noise), extracted from final analysis, components with spatial characteristics comprising individual functional networks should be grouped together (Figure 8). Final RSN maps are typically presented orthogonally (i.e., in representative sagittal, coronal, and axial views), although components can also be shown displayed in serial axial sections for completeness (Figure 8), as described above (see How to Visualize Components). Detailed descriptions of brain regions comprising each brain network are beyond the scope of this manuscript. Ultimately, meaningful evaluation and interpretation of individual and group-level networks requires a thorough understanding of previously reported RSNs, and the brain regions they encompass. For spatial maps and descriptions of anatomical structures comprising individual networks, please refer to the published literature referenced in Section “Review of Current Literature on Resting-State Networks in Infants” of the *Discussion*.

## Discussion

Despite its complexities and shortcomings, rs-fMRI is undoubtedly a valuable tool for gaining insights into brain function. As such, rs-fMRI represents a relatively new neuroinvestigative tool for probing functional architecture in the infant brain. While investigations to-date yield promising results, this rapidly emerging field is faced with several methodological considerations (discussed below) that will need to be refined prior to standardization of the method across research and clinical fields.

### Methodological Challenges

Despite significant advances since its inception, expanded applications of rs-fMRI to infant populations raises many methodological concerns that have yet to be resolved, including systematic, age-specific preprocessing techniques and parameters for analyzing rs-fMRI data.

#### Age-Specific Atlases

The infant brain undergoes rapid postnatal brain development, involving subject- and region-specific alterations in cortical density, thickness, and folding, such that visible changes can be seen from week to week (78–80). Misclassifications and biases can result from the use of inappropriate templates during registration (96), compromising specificity of alignment. Registration of each functional image to its native space (i.e., corresponding structural T1 image) accounts for individual morphological variability and age-dependent differences, allowing for more accurate spatial normalization and heightened sensitivity of signal detection in activated brain regions. However, it is one of the most time consuming steps due to the lack of reliable neonatal segmentation techniques, necessitating manual editing. Novel neonatal brain extraction methods continue to emerge (97, 98), including some algorithms insensitive to brain pathology (99). In studies that handle larger cohorts of infants, it may be more practical to register subjects’ fMRI images to a common stereotactic space. As previously mentioned, the latter is absolutely required for intersubject statistical comparison using GICA. Methods to create study-specific pediatric MRI average brain templates are available (100, 101). Alternatively, standardized age-specific templates and atlases are becoming more readily available (15, 80, 102–104). However, it is imperative that users understand the origins and assumptions made in creating such templates. Relevant concerns regarding preexisting templates and atlases include: (1) averages arising from a limited number of subjects [e.g., seven infants in one study (80, 105)], (2) using non-generalizable clinical populations [e.g., sedated infants scanned for clinical indications (103)], and/or (3) using broad age ranges unable to appreciate regional postnatal neurodevelopmental changes [e.g., 1-year-old template derived from 9- to 15-month-old infants (103)]. Furthermore, a predominance of templates to date, are compiled for specific and/or widely spaced time points across early development (typically term-equivalent age, 1- and 2-year-old infants) (80, 100, 104, 106). Recent movements toward establishing comprehensive, freely available MRI databases show promise. Notably, the publicly available Neurodevelopmental MRI Database offers age-specific templates at narrow age-windows (1–3 month intervals) throughout early infancy, as well as reference templates from early childhood through to early adulthood (6-month increments)^19^ (101). However, at this early stage, infant templates are derived from limited number of subjects and represent an average of healthy infant patient populations, specifically recruited as normally developing or control subjects. Currently, no robust age-specific infant atlases (in months) akin, for example, to the MNI152 in adults are freely available for systematic use in infants. Future efforts should be directed toward creation of freely available standardized age- and population-specific atlases for infants that would allow for more accurate registration and comparative data analyses necessary for longitudinal studies of infant brain development.

#### Motion

Motion is a prominent concern in the field of rs-fMRI, particularly in investigations of infant and clinical populations. Emerging evidence suggests even subtle head motion can introduce systematic biasing effects on measures of network connectivity, including distance-dependent modulation of correlation (51, 107–110). General consensus in resting-state literature holds that proper analysis of intrinsic brain activity requires at least ~5 min of temporally contiguous data to remain post motion scrubbing (41, 51). This presents a major problem for infants, where data quality hinges upon an infant remaining perfectly still for the duration of scan. Infant motion is observed during wakefulness, as well as during sleep. MRI machines are loud and transitions between sequences often startle the infant, contributing to sleep difficulties. Moreover, individual differences (i.e., age, disposition, health) may impact successful scan completion. Numerous strategies have been adopted to attenuate scanner noise and promote minimal subject motion, including the use of consistent background noise (to acclimate infant) combined with neonatal earmuffs or foam earplugs, catering to infant’s circadian rhythm (i.e., scanning at night), and swaddling infant prior to scan (18, 111). Recently developed protocols show promise in mitigating the incidence of infant motion, reporting higher rates of scan completion and structural scan acquisition sans motion artifact, as well as amelioration of SNR (112, 113). Further refinement of scan acquisition is critical moving beyond establishment of normative resting-state patterns, to understand complex phenomenon such as brain development across decades and abnormal processes in clinical populations. Improved techniques for reliable post-acquisition detection and elimination of motion artifact also remain an active field of investigation.

#### Level of Arousal

Studies of infant populations demonstrated the ability to detect RSNs during sleep and sedation. However, targeted investigations probing the effect of level of arousal (e.g., sleep stages) on functional connectivity in infants have yet to be published. Limited studies addressing this topic report minimal effects of sleep on baseline BOLD signal (114, 115). Currently, methodological limitations prevent simultaneous use of electroencephalogram during rs-fMRI, which could ideally be used to track and control for various sleep stages (116). An alternative strategy to control for sleep stage entails fixing order of scan sequences, such that rs-fMRI data are acquired first, immediately after infants fall asleep (116). RSNs have been identified during scans using light sedation (22, 25, 32, 117–119), with several studies reporting no significant discrepancies in qualitative or quantitative results between infants scanned with and without sedation (25, 27, 87). However, past studies in adults cited state-dependent modulation of correlation strength in a network-specific pattern (120–122). While sedation is particularly attractive for investigations in infant populations (to reduce motion), the effect of sedation on RSNs remains controversial and incompletely understood. Furthermore, in light of potential detrimental effects of sedation and anesthetic drugs on infant brain development (123–125), it may be unethical to consider such measures for research purposes.

#### Frequency Characteristics

Since the first observation of low-frequency RSNs (7), model-based strategies for identification and removal of artifacts have been developed based on adult physiology. Results of these investigations have been generalized to infant resting-state dynamics, guiding parameter choice despite notable differences in physiology (105). Infants typically exhibit higher baseline cardiac and respiratory rates, as well as increased variability in tidal volume and measures of heart rate. Systematic integration of subject monitoring for such physiological data has not been routinely collected during past rs-fMRI investigations (105). Simultaneous physiological monitoring during fMRI scan acquisition is associated with many technical challenges, chiefly related to ensuring the safety of subject, operator, and equipment (126). This information may help to define age-specific models of physiologic noise, allowing for targeted removal of artifact (physiologic and motion-related) embedded in infant data, as well as characterization of RSN spectral features and selection of appropriate preprocessing parameters.

#### Ethical Considerations

In addition to the challenges posed by infant RSN analysis, many ethical issues arise when conducting research in human infants. These include obtaining informed consent from caregivers from diverse educational and cultural backgrounds, as well as comprehensive pre-screening of caregivers and physicians with pertinent knowledge of patient’s medical history to provide for any fMRI contraindications (127, 128). Apart from general safety measures, additional infant-specific considerations involve curtailing infant distress that may result from the fMRI protocol. Most neuroimaging of healthy infants is performed during natural sleep without sedation for a number of reasons, including safety, practicality, and parental comfort. As a result, infants often awaken during scan acquisition and may experience stress due to novel scanner environment (e.g., loud noise). Protocols tailored to safeguard against infant distress involve careful monitoring of infant wakefulness, with contingency plans in place to quickly respond to symptoms of distress (21).

#### Shortcomings of ICA Approach

Independent component analysis provides blind statistical processing of rs-fMRI data, facilitating data-driven exploratory analysis important in instances where no suitable hypothesis is available. Further, it helpfully segregates noise from valuable neural signal during decomposition process, providing several opportunities to remove artifact during preprocessing and final analysis. However, ICA-based artifact removal does not ensure elimination of all noise from the data, and ICA is potentially more sensitive to influences of non-neuronal signal than other methods (44). Moreover, ICA data decomposition ultimately compels manual selection of important components reflecting true brain networks from artifactual derivatives. While considered the gold standard (129–131), manual classification is time consuming and requires expertise, including a robust understanding of infant physiology and MR physics critical for informed evaluation of components (74). Fundamental advancements in fully automated approaches to component classification (e.g., FIX classifier) have been developed and demonstrate high accuracies in adult analyses. However, such methods require further refinement for robust and reliable application in infant populations, including integration of age-specific modeling of physiologic noise.

### Review of Current Literature on RSNs in Infants

Application of rs-fMRI in infants is a relatively recent phenomenon, and the literature is correspondingly sparse. The first ever report of infant RSNs described a total of five networks in former premature infants scanned at term-equivalent age (estimated 40 weeks gestational age) (22). Since then, the majority of studies sought to define normative neural network development at specific developmental time points in healthy preterm and term infants. Fragmented precursors of RSNs have been detected as early as 26 weeks postmenstrual age (33), with all major adult RSNs present to some degree at term (25). Primordial RSNs in the youngest subjects are often described as “local blooms” (i.e., network spatial diffusivity), whereas older infants exhibit more focal brain activity (33). This transitional trend towards spatial localization is thought to reflect maturation of structural and functional organization of the brain with advancing age. Younger infants around term-equivalent age tend to exhibit stronger local connections and interhemispheric connectivity between homogeneous counterparts (22), as well as weaker long-range and intrahemispheric connectivity between disparate regions (22, 33). The common consensus is that gradual network maturation occurs with advancing age. Increasing postmenstrual age has been associated with increased connectivity strength, including connections between physically distant brain regions (132). Indeed this rapid increase in connection density peaks by the first year of life, after which it begins to stabilize (132). Cortical hubs (unusually interconnected brain regions, thought to play an integral role in information flow) identified in the infant brain typically encompass primary sensory systems (e.g., auditory, visual, sensorimotor), as opposed to higher association cortices associated with default-mode and frontoparietal attentional networks in adults (22, 133, 134). This finding is supported by longitudinal investigations, which report network-specific rates of maturation with functional connections necessary for higher order cognitive functions appearing later in development (25). While resting-state literature to date focuses on healthy infant populations (i.e., without apparent clinical problems), prematurity itself engenders increased risk of various poor sequela, including cognitive and learning deficits (135). In one study, preterm infants exhibited weaker functional connectivity than term infants in a network-specific pattern that became more distinct over time (87). While some studies report disparities in RSN development between preterm and term infants (33, 87), study by Doria et al. (25) did not find such evidence. Insufficient literature addressing the differences between preterm and term infant populations precludes any assertions one way or another at this time. Further, the vast majority of these studies included only healthy preterm infants, with no signs of overt brain lesions or neurodevelopmental impairment. Such methods may introduce some degree of bias in that comparisons to date have not been conducted using a truly representative cross-section of healthy preterm infants. Published reviews on infant rs-fMRI continue to synthesize findings to date, important for propelling the field forward (21, 72, 105, 116).

### Future Applications of Resting-State Analysis in Infants

#### Understanding Neurodevelopment and Neuroplastic Vulnerability

Resting-state functional magnetic resonance imaging provides insight into intrinsic functional organization of the brain, implicated in development of normative stimulus response and longitudinal neurobehavioral outcomes (136). Both deprivation and overabundance of sensory stimuli introduced during critical periods of development are known to induce physiological and structural changes that alter brain circuitry (137, 138). While such neuroplasticity endows a certain degree of productive adaptability, it also renders the developing brain highly vulnerable to any number of environmental stressors. Past research using functional near-infrared spectroscopy and electroencephalogram in preterm infants observed pain processing involving cortical structures as early as 25 weeks postmenstrual age (139, 140), as well as hypersensitivity to painful stimuli when compared to term infants (141). Furthermore, studies of preterm infants in the NICU found total number of medical procedures predicted regional structural and functional alterations at term-equivalent age (94). However, the underlying neural mechanisms and encompassing brain regions responsible for such changes remain unknown. Rs-fMRI has the potential to elucidate immediate and long-term neural sequelae of events including premature birth, perinatal pain exposure (e.g., procedural), and drug treatment. Future investigations in critically ill infant populations may provide insight into critical factors that determine neurodevelopmental outcome, guiding targeted preventative strategies to mitigate risk factors and development of early intervention therapeutics. Given the rapidity of postnatal neurodevelopment and importance in timing of exposure to environmental stressors, follow-up studies tracking infant developmental progression, together with long-term imaging and behavioral outcomes, are integral to establishing biomarkers with true predictive value.

#### Clinical Applications

Resting-state functional magnetic resonance imaging holds unique advantages over task-based fMRI, allowing for investigations of wider range of patient populations and elimination of numerous experimental confounds (142–144). In adults, early clinical investigations of RSNs centered on patient populations with neuropsychiatric disorders (12, 144, 145), laying the groundwork for expanded inquiries aimed at diagnosis, treatment efficacy, and longitudinal tracking of disease course. Thus far, these studies demonstrate patterns of decreased connectivity strength across numerous RSNs in patients that correlate with disease progression in certain clinical populations, including Alzheimer’s disease, Parkinson’s disease, multiple sclerosis, Tourette’s syndrome, and autism disorder (12, 145, 146). Diagnostic value of rs-fMRI has also proven attractive for application in disorders of consciousness (e.g., coma, brain death), which currently present a major clinical challenge associated with a high rate of misdiagnosis (40%) (6, 147). The utility of rs-fMRI in infant populations represents a largely untapped opportunity to advance clinical research in a number of areas. Notably, recent investigations into the etiology of autism successfully implemented infant rs-fMRI to uncover early biological markers of the disorder, as well as modeled its neurodevelopmental trajectory (148, 149). Converging evidence from diverse modalities, including structural MRI and diffusion tensor imaging, is critical for empowering more robust interrogations of pathological/atypical processes. The potential for discovery using rs-fMRI in infant populations is considerable and will hinge on future technological advances to drive its application into routine clinical practice.

## Ethics Statement

This study was approved by Boston Children’s Hospital Institu-tional Review Board (IRB-P000007855). Informed consents for all subjects were obtained in accordance with the Declaration of Helsinki.

## Author Contributions

Authorship credit was based on substantial contributions to (1) the conception and manuscript design (CM and DBa); (2) acquisition and analysis of data, or interpretation of data (CM, RJ, LB, and DBa); (3) drafting the article or revising it critically for important intellectual content (CM, DBo, LB, and DBa); (4) final approval of the version to be published (CM, RJ, DBo, LB, and DBa); and (5) are accountable for all aspects of the work in ensuring that questions related to the accuracy or integrity of any part of the work are appropriately investigated and resolved (CM, RJ, DBo, LB, and DBa).

## Conflict of Interest Statement

None of the authors have any conflict of interest, including specific commercial or financial interests, relationships or affiliations relevant to the manuscript that could be construed as a potential conflict of interest.

## Footnotes

^1^http://www.mathworks.com/products/matlab.html.

^2^http://people.cas.sc.edu/rorden/mricron/index.html.

^3^http://fsl.fmrib.ox.ac.uk/fsl/fslwiki/FslInstallation.

^4^http://fsl.fmrib.ox.ac.uk/fsl/fslwiki/MELODIC.

^5^http://mialab.mrn.org/software/gift/.

^6^MCFLIRT stands for Motion Correction by FMRIB’s Linear Image Registration Tool.

^7^https://fsl.fmrib.ox.ac.uk/fsl/fslwiki/FNIRT.

^8^Ensemble learning (i.e. “stacking”) refers to the strategic use of multiple classifiers to enhance accuracy of the overarching predictive model, compensating for weakness in any one algorithm through the strengths of others (61).

^9^https://fsl.fmrib.ox.ac.uk/fsl/fslwiki/FIX/UserGuide#Training_datasets.

^10^https://fsl.fmrib.ox.ac.uk/fsl/fslwiki/MELODIC#Using_melodic_for_just_doing_mixture-modeling.

^11^Full width at half maximum (FWHM) refers to the difference between the two extreme values; it is the width of a spectrum curve measured between extreme points on the *y*-axis, which are half the maximum amplitude.

^12^https://fsl.fmrib.ox.ac.uk/fsl/fslwiki/GLM.

^13^http://fsl.fmrib.ox.ac.uk/fsl/fslwiki/Melview.

^14^https://fsl.fmrib.ox.ac.uk/fsl/fslwiki/FSLeyes.

^15^http://www.humanconnectome.org/software/get-connectome-workbench.

^16^https://afni.nimh.nih.gov/afni.

^17^http://www.mccauslandcenter.sc.edu/mricro/mricron/.

^18^Spatial maps derived from final analysis (spatially smoothed) will look different from those observed after the first run of ICA during preprocessing (spatially unsmoothed), which typically exhibit noisier patterns of activation clusters.

^19^http://jerlab.psych.sc.edu/NeurodevelopmentalMRIDatabase.

## Abbreviations

AFNI, Analysis of Functional NeuroImages; BET, Brain Extraction Tool; BIRN, Biomedical Informatics Research Network; BOLD, bold oxygen level dependent; CSF, cerebrospinal fluid; DVARS, derivative of root mean squared variance over voxels; EPI, echo-planar imaging; FAST, FMRIB’s Automated Segmentation Tool; FEAT, FMRI Expert Analysis Tool; FIX, FMRIB’s ICA-based X-noisifier; FLIRT, FMRIB’s Linear Image Registration Tool; fMRI, functional magnetic resonance imaging; FNIRT, FMRIB’s Non-linear Image Registration Tool; FSL, FMRIB Software Library; FWHM, full width at half maximum; GICA, group independent component analysis; GIFT, Group ICA of fMRI Toolbox; HDR, high dynamic range; IC, independent component; ICA, independent component analysis; ICA-AROMA, ICA-based Strategy for Automatic Removal of Motion Artifacts; MCFLIRT, Motion Correction by FMRIB’s Linear Image Registration Tool; MELODIC, Multivariate Exploratory Linear Decomposition into Independent Components; MNI, Montreal Neurological Institute; MPRAGE, magnetization prepared rapid acquisition gradient echo; MRI, magnetic resonance imaging; PCA, principal component analysis; PICA, probabilistic independent component analysis; RMS, root mean squared; ROI, region-of-interest; rs-fMRI, resting-state functional magnetic resonance imaging; RSN, resting-state network(s); SCA, seed-based correlation analysis; SNR, signal-to-noise ratio; TE, echo time; TR, repetition time.
